# Forty‐three key gene expressions involved in the effect of indoleamine 2,3‐dioxygenase 1 expression on cancer prognosis may be a potential indoleamine 2,3‐dioxygenase 1 inhibitor biomarker

**DOI:** 10.1002/ctm2.330

**Published:** 2021-02-17

**Authors:** Weirui Li, Leilei Guo, Zikang Xing, Xin Fang, Heng Liang, Shengnan Zhang, Lei Shi, Chunxiang Kuang, Leming Shi, Yuanting Zheng, Yueqing Hu, Qing Yang

**Affiliations:** ^1^ State Key Laboratory of Genetic Engineering School of Life Sciences Fudan University Shanghai China; ^2^ Shanghai Key Lab of Chemical Assessment and Sustainability School of Chemical Science and Engineering Tongji University Shanghai China; ^3^ Shanghai Center for Mathematical Sciences Fudan University Shanghai China

Dear Editor,

Indoleamine 2,3‐dioxygenase 1 (IDO1) inhibition has been developed as a potential new tool in cancer immunotherapy and some IDO1 inhibitors have been in clinical trials.^1^ However, the biomarker information of IDO1 inhibitors is very few. Here, we searched for potential IDO1 inhibitors biomarker by identifying molecular characteristic that can predict the effect of IDO1 on cancer prognosis. We evaluated the effect of *IDO1* mRNA expression on prognosis in 33 diverse cancer types, identified 43 key genes involved in the effect, and defined the weighted average of all the 43 key gene expressions as 43‐gene score to reflect the integrated role of 43 key gene expressions in the relationship between *IDO1* expression and cancer prognosis. We mined the potential regulator of 43 key genes and explored its impacts on regulating the expressions of 43 key genes and enhancing the therapeutic efficacy of IDO1 inhibitor (Figure 1).

**FIGURE 1:**
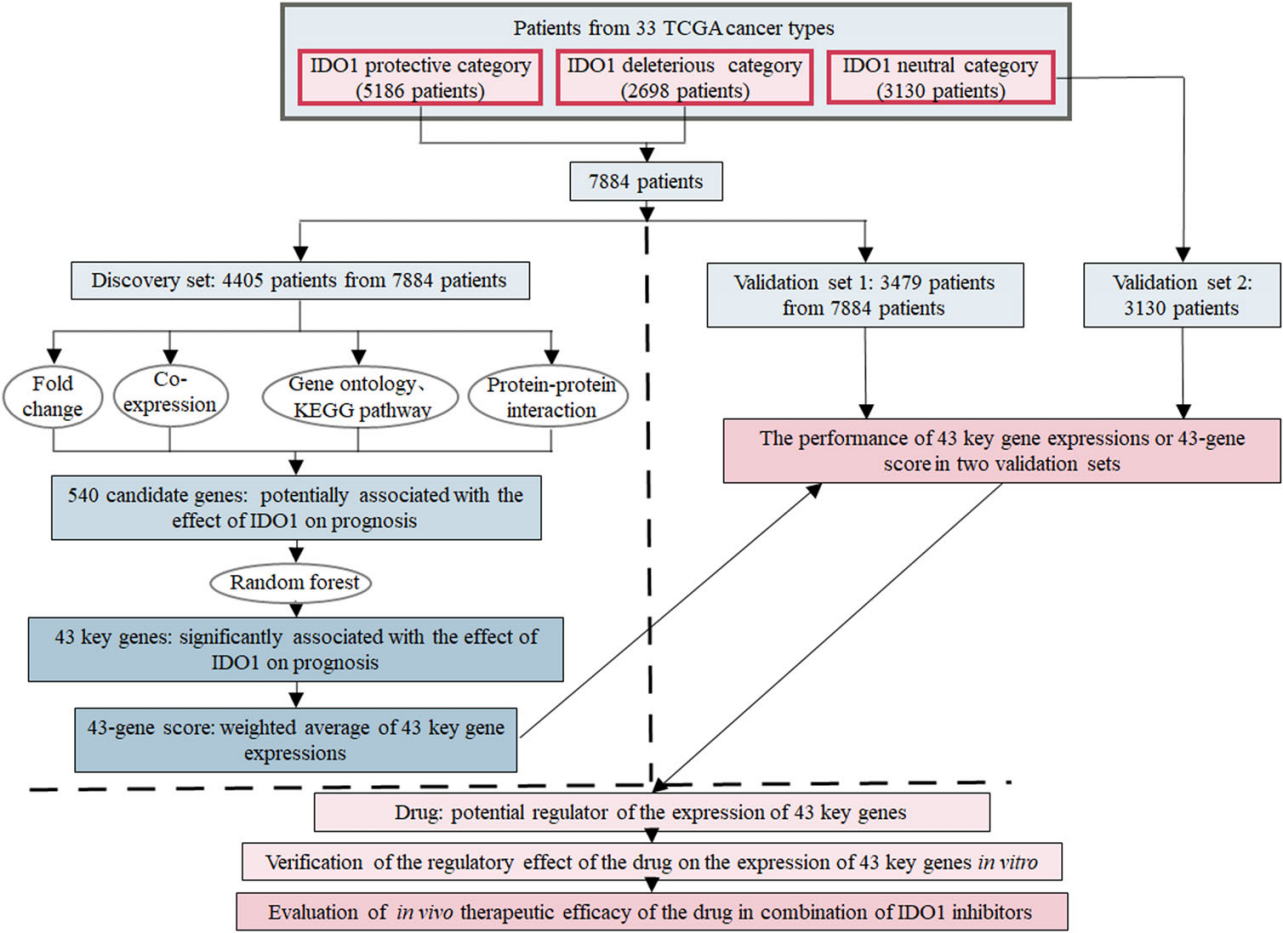
Study flowchart. A total of 43 key genes influencing the association between *IDO1* expression and prognosis were identified based on 4405 patients in the discovery set and evaluated in 3479 and 3130 patients in the validation sets 1 and 2, respectively, from TCGA Pan‐Cancer dataset. Protective, *IDO1* high expression correlates to good prognosis; Deleterious, *IDO1* high expression correlates to poor prognosis; Neutral, *IDO1* expression does not correlate to the prognosis; TCGA, The Cancer Genome Atlas; KEGG, Kyoto Encyclopedia of Genes and Genomes

Specifically, we used Kaplan‐Meier method and Cox proportional hazards (CoxPH) regression model to compare the overall survival (OS) and progression‐free interval (PFI) of patients with high and low *IDO1* expressions^2^, ^3^ (Table S1) and evaluated the effects of *IDO1* expression on prognosis in 33 diverse cancer types from The Cancer Genome Atlas (TCGA) (Figure S1). These types of cancer were then classified into IDO1 protective category, in which *IDO1* has beneficial effects on prognosis, IDO1 deleterious category, in which *IDO1* has disadvantageous effects on prognosis, and IDO1 neutral category, in which *IDO1* has no effects on prognosis (see Table S2 for more details). From the discovery set, which is a random partition of patients of protective and deleterious categories, computational tools such as the detection of differentially expressed genes (DEGs),^4^ weighted gene co‐expression network analysis (WGCNA)^5^ (Figure 2A; Figures S2‐S6), gene set enrichment analysis (GSEA)^6^ (Figure 2B,C) and protein‐protein interaction network (PPIN) analysis^7^ (Figure 2D) were employed to obtain five candidate gene sets. Totally, 540 candidate genes associated with the effects of *IDO1* expression on prognosis were collected and shown in Table S3. Based on a random‐forest variable importance measure, these genes were further refined to 43 key genes (Table S4) and 43‐gene score. The 43‐gene scores were significantly lower in IDO1 deleterious category than that in IDO1 protective category (Figure 2E). The effects of *IDO1* on prognosis were more significant in two subgroups separated by the median of 43‐gene scores than that in the whole discovery set (Figure 2F). We also got the similar observation in the validation set 1 (Figure 2G). For the patients in the discovery set and the validation set 1, 43‐gene score showed superior prediction power, especially over the patient category (Figure 2H). Within our expectation, the performance of 43‐gene score was not strong in the validation set 2, in which all the patients are of the IDO1 neutral category (Figure S7). Even so, we observed that some key genes, such as glial fibrillary acidic protein (*GFAP*) and X‐C motif chemokine ligand 1 (*XCL1*), still performed well in the validation set 2 (Figure 2I). All of these illustrated that 43‐gene score is a better biomarker than the patient category regarding the efficacy of IDO1 inhibitors and may also reflect the immune landscape in tumor.

**FIGURE 2:**
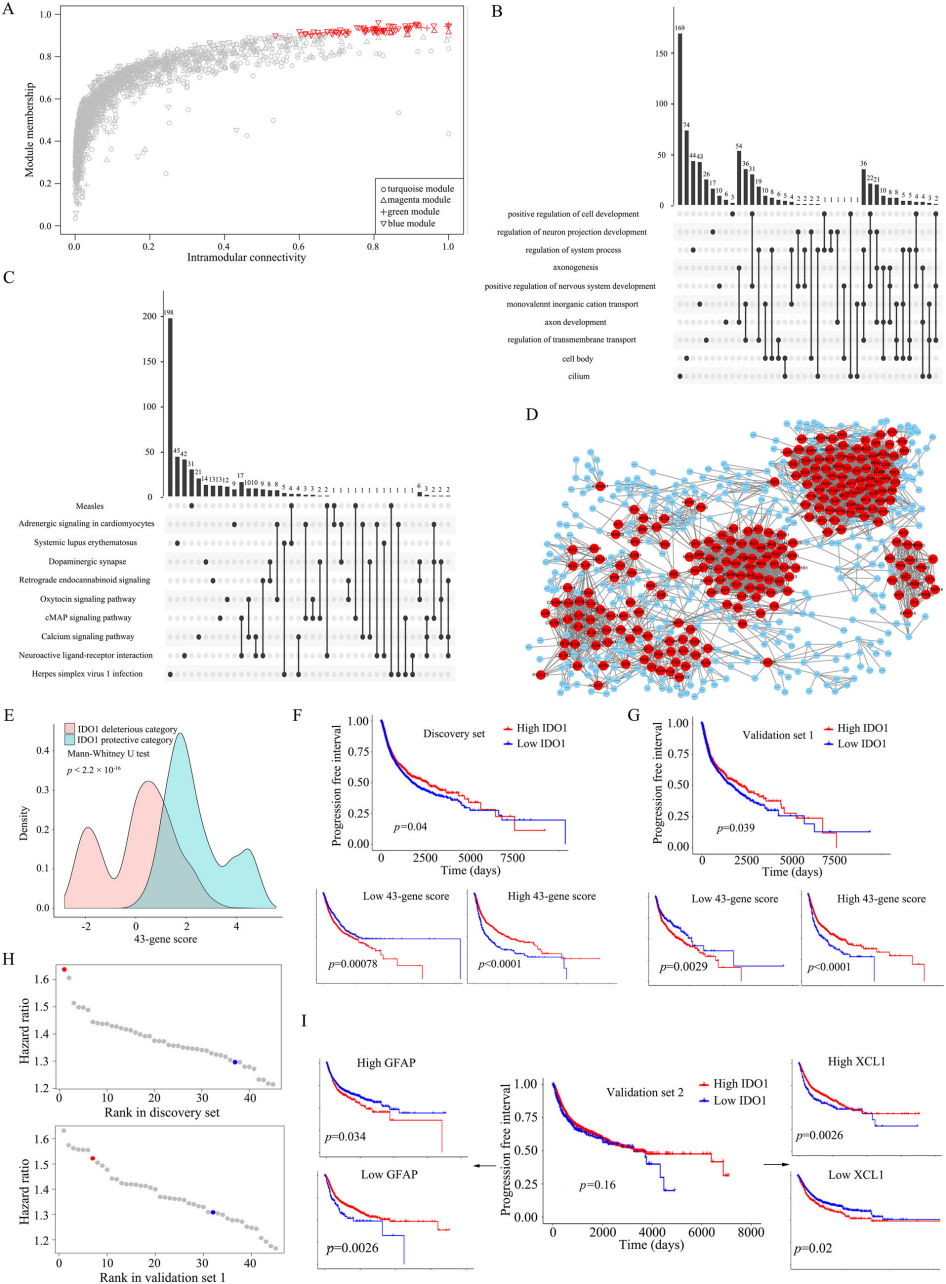
Candidate genes according to expression features and biological functions and the performance of key genes in predicting the effect of *IDO1* on prognosis. A, The scatterplot of module membership against intramodular connectivity of genes in the turquoise, magenta, green and blue modules from WGCNA. The module membership (MM) of gene is defined as the correlation of expression profile and each module eigengene. The intramodular connectivity of gene measures how connected, or co‐expressed, a given gene is with respect to the genes of a particular module. Candidate genes were highlighted in red. B,C, The upset plots showing the overlapping of leading edge genes among top 10 enriched GO terms and KEGG pathways from GSEA. Unconnected dots are leading edge genes with only one enriched GO term or KEGG pathway. Connected dots indicate leading edge genes shared by two or more enriched GO terms and KEGG pathways. The vertical histogram shows the number of overlapping leading edge genes. D, Protein–protein interaction network of DEGs with |log_2_(FC)| > 1.5. The nodes represent proteins coded by DEGs with |log_2_(FC)| > 1.5. The edges represent interactions between proteins. Interactions were retrieved from STRING online database. Interactions with scores greater than 0.9 were retained. Proteins encoded by candidate genes were highlighted in red. E, Distribution of 43‐gene scores of IDO1 deleterious category patients (pink) versus that of IDO1 protective category patients (blue) in the discovery set. The *P*‐value was calculated by Mann‐Whitney *U* test. F,G, Kaplan‐Meier curves of the progression‐free interval stratified by low (in blue) and high (in red) expression of *IDO1* for the patients in the discovery set (top) and validation set 1 (top), and further their subgroups of low (bottom left) or high (bottom right) 43‐gene score, respectively. The median of 43‐gene scores was set as the cutoff value for determining whether a patient has a high or low 43 gene score. The p‐values were calculated by the log‐rank test. The C‐indexes in terms of high or low IDO1 expression for the discovery set and validation set 1 are 0.519 and 0.527, respectively. When each of these two sets are partitioned into two subsets according to high or low 43‐gene scores, the C‐indexes increase to 0.623 and 0.571 for discovery set, and 0.607 and 0.580 for validation set 1, respectively. H, The ranking of the hazard ratios of each of the 43 key gene expressions (in grey), 43‐gene score (in red), and patient category (in blue) in the discovery set (upper) and validation set 1 (bottom), respectively. Clinical endpoint is the progression‐free interval. I, Kaplan‐Meier curves of the progression‐free interval stratified by low (in blue) and high (in red) expression of *IDO1* for the patients in the validation set 2 (middle). Further, Kaplan‐Meier curves for patients with high (upper left) and low (bottom left) *GFAP* expression, and for patients with high (upper right) or low (bottom right) *XCL1* expression. The median of *GFAP* or *XCL1* expression was set as the cutoff value. *P*‐values were calculated by log‐rank test. WGCNA, weighted gene co‐expression network analysis; GO, Gene Ontology; KEGG, Kyoto Encyclopedia of Genes and Genomes; GSEA: gene set enrichment analysis; DEGs, differentially expressed genes; FC, fold change. STRING, Search Tool for the Retrieval of Interacting Genes/Proteins

We further found that the tumor immune subtype was distributed differently among patients of IDO1 protective, deleterious, and neutral categories (Figure S3). *IDO1* had a beneficial effect on prognosis in patients with tumor immune subtype C1 (Wound Healing) or C2 (IFN‐gamma Dominant), a disadvantageous effect in patients with C3 (Inflammatory) or C5 (Immunologically Quiet) (Figure S4). We identified four co‐expression modules that were associated with tumor immune subtype and patient category based on their respective correlations with module eigengenes of modules (Figure S5). The correlations between gene significance (GS) and module membership (MM) in the four modules were illustrated in Figure S6, which confirmed that the larger the MM of a gene was, the stronger the correlation between the gene and immune subtype or patient category was. Tumors with C1 or C2 have the highest proportion of tumor infiltrating lymphocytes and tumors with C3 or C5 have the opposite.^8^ It can be concluded that IDO1 inhibitors are applicable for the patients with C3 or C5 because *IDO1* has a disadvantageous effect on their prognoses. We also found that tumor immune subtype's distributions were significantly different in patients with high and low *GFAP* or *XCL1* expressions and in patients with high and low 43‐gene scores (Figure S8). These suggest that *GFAP* and *XCL1* may be the more important ones among the 43 key genes.

We sought to find the potential regulator of the expressions of 43 key genes using the Genomics of Drug Sensitivity in Cancer database. As *IDO1* high expression correlates to good prognosis in patients with high 43‐gene scores, a hierarchical logistic regression model was employed to fit the efficacy measures of 251 drugs across 983 human cell lines, and further to identify drugs more sensitive to that cell lines with higher 43‐gene scores. As a result, EGFR inhibitor gefitinib was mined (Figure 3A‐E). Using LLC cells and LLC tumor‐bearing mouse model,^9^ the effect of gefitinib in regulating expressions of key genes (Figure 3F,I) and enhancing the therapeutic efficacy of IDO1 inhibitor (INCB024360, L‐1‐MT, and RY103) were explored (Figures 3G,H,J), which supports the possibility of key gene expressions as a biomarker for IDO1 inhibitors. To exhibit the stability of our procedure for obtaining key genes, we randomly partitioned the patients in either the IDO1 protective or deleterious category into a new discovery set and a new validation set 1, and then repeated the same steps and finally got the similar results (see details in Tables S5 and S6 and Figure S9.

**FIGURE 3:**
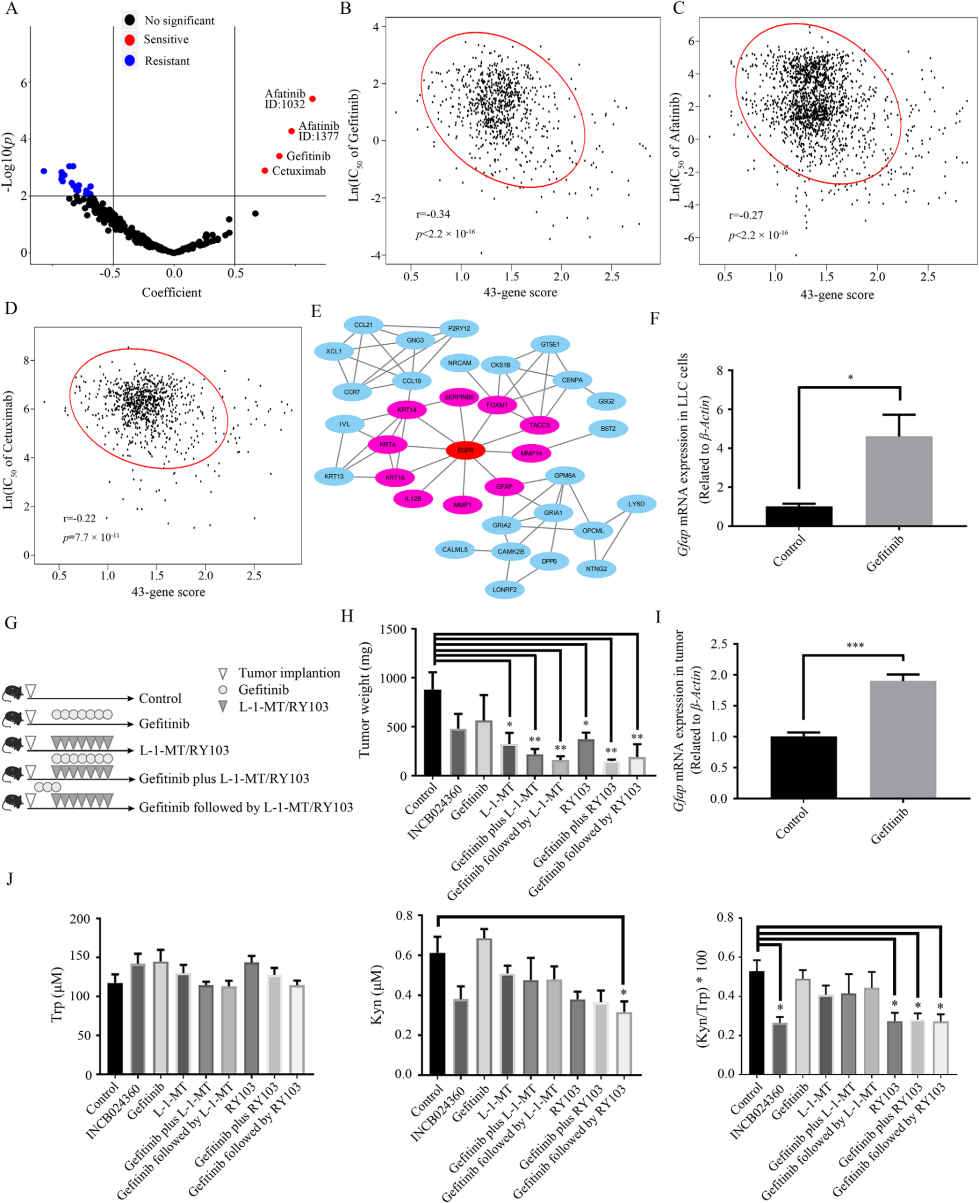
Gefitinib, an EGFR inhibitor being a potential regulator of the expressions of 43 key genes, enhanced the anti‐tumor effect of IDO1 inhibitor in vivo. A, Volcano plot showing the coefficients and *p*‐values from the hierarchical logistic regression model fitting IC_50_ of 251 drugs and 43‐gene scores of 983 cell lines. Each point is an indicator of drug and the significance level was 0.01. The coefficient reflects the change of drug sensitivity with 43‐gene score in a cell line. The positive coefficient indicates that cell lines with higher 43‐gene scores were more sensitive to this drug. B‐D, The scatterplots of ln(IC_50_) of gefitinib (B), afatinib (C), cetuximab (D) against 43‐gene score with 95% confidence ellipse, Pearson correlation coefficient, and its corresponding *P*‐value. E, Protein–protein interaction network of EGFR and 43 key genes. The interactions were retrieved from STRING online database and shown as edges when the scores are greater than 0.4. Protein with no edges connected with other proteins were hidden. The proteins interacting with EGFR were marked in pink. F, qPCR detection of changes in the mRNA expression of *Gfap* in LLC cells due to the treatment of gefitinib (100 nM, 96 h). Control group received equal amount of DMSO for dissolving gefitinib. Results are representative of at least three independent experiments. G, *In vivo* study design. H, Weight of tumors in mice at the time of sacrifice from each of nine groups. I, qPCR detection for mRNA expression of *Gfap* in tumors from control and gefitinib administrated mice. J, Concentration of Trp (left) and Kyn (middle) and their ratio Kyn/Trp (right) in serum from each group by HPLC. Data are represented as mean ± SEM. Control: n = 5; INCB024360: n = 5; Gefitinib: n = 5; L‐1‐MT: n = 6; Gefitinib plus L‐1‐MT: n = 6; Gefitinib followed by L‐1‐MT: n = 6; RY103: n = 6; Gefitinib plus RY103: n = 5; Gefitinib followed by RY103: n = 6. *P*‐values were calculated by Student's t‐test or one‐way ANOVA followed by Dunnett's test. *.01 < *P‐*value < .05, ** .001 *<* *P‐*value < .01, *** *P‐*value < .001

Our study provides a possible strategy for the screening of IDO1 inhibitor biomarker and suggests a new therapeutic strategy to enhance the therapeutic efficacy of IDO1 inhibitors.

## AUTHOR CONTRIBUTIONS

Qing Yang, Yuanting Zheng, Yueqing Hu, and Weirui Li conceived and designed the study. Weirui Li processed the data and led the bioinformatics analysis. Weirui Li, Leilei Guo, Zikang Xing, Xin Fang, Heng liang, Shengnan Zhang, and Lei Shi performed the qPCR, HPLC analysis, and the animal experiments. Chunxiang Kuang designed and synthesized the IDO1 inhibitor RY103. Qing Yang, Yueqing Hu, and Weirui Li wrote the manuscript. Qing Yang, Yuanting Zheng, Yueqing Hu, and Leming Shi supervised the project. All the authors contributed to the critical revision of the manuscript and approved the final version.

## FUNDING INFORMATION

This work was supported by the Key Biomedical Program of Shanghai (NO. 18431902600 &17431902200) and the National Natural Science Foundation of China (NO. 11971117).

## DATA AVAILABILITY STATEMENT

The datasets used or analyzed in this study are available from the corresponding author on reasonable request.

## CONSENT FOR PUBLICATION

All the authors consent for publication.

## CONFLICT OF INTEREST

The authors declare no conflict of interest.

## Supporting information



SuppMat1

SuppMat2

SuppMat3
