# Supplementary material for: Forty‐three key gene expressions involved in the effect of indoleamine 2,3‐dioxygenase 1 expression on cancer prognosis may be a potential indoleamine 2,3‐dioxygenase 1 inhibitor biomarker
Source: Clin Transl Med. 2021 Feb 17;11(2):e330. doi: 10.1002/ctm2.330 (PMC7888544; doi:10.1002/ctm2.330)
Supplement: Supplementary file 1 — SuppMat1 [file CTM2-11-e330-s001.docx]

**Supporting Information 1:** Supplementary tables

**43 key gene expressions involved in the effect of indoleamine 2,3-dioxygenase 1 (IDO1) expression on cancer prognosis may be a potential IDO1 inhibitor biomarker**

**Authors:** Weirui Li^1^, Leilei Guo^1^, Zikang Xing^1^, Xin Fang^1^, Heng liang^1^, Shengnan Zhang^1^, Lei Shi^1^, Chunxiang Kuang^2^, Leming Shi^1^, Yuanting Zheng^1^*, Yueqing Hu^1, 3^*, Qing Yang^1^*

*Corresponding author

**Author’s institutional affiliations:**

^1^ State Key Laboratory of Genetic Engineering, School of Life Sciences, Fudan University, Songhu Road 2005, Shanghai, 200438, China

^2^ Shanghai Key Lab of Chemical Assessment and Sustainability, School of Chemical Science and Engineering, Tongji University, 1239 Siping Road, 200092, Shanghai, China

^3^ Shanghai Center for Mathematical Sciences, Fudan University, Shanghai, China

Supplementary tables

**Table S1. Results of association study between *IDO1* expression and OS or PFI in 33 cancer types.**

| **TCGA code** | **N** | **OS** | | |  | **PFI** | | | |
| --- | --- | --- | --- | --- | --- | --- | --- | --- | --- |
|  |  | ***p*-value in KM analysis** | ***p*-value in CoxPH regression** | **Significant** |  | ***p*-value in KM analysis** | ***p*-value in CoxPH regression** | **Significant** | |
| ACC | 92 | 0.24316 | 0.24904 | no |  | 0.03634 | 0.06865 | * |  |
| BLCA | 412 | 0.01948 | 0.02050 | * |  | 0.03861 | 0.03951 | * |  |
| BRCA | 1097 | 0.00122 | 0.00168 | ** |  | 0.00235 | 0.00259 | ** |  |
| CESC | 307 | 0.02932 | 0.03373 | * |  | 0.00081 | 0.00242 | ** |  |
| CHOL | 45 | 0.02754 | 0.03304 | * |  | 0.00021 | 0.00080 | *** |  |
| COAD | 459 | 0.02324 | 0.02497 | * |  | 0.00115 | 0.00135 | ** |  |
| DLBC | 48 | NA | NA | NA |  | 0.05769 | 0.07355 | no |  |
| ESCA | 185 | 0.01092 | 0.01272 | * |  | 0.14378 | 0.14653 | no |  |
| GBM | 596 | 0.05679 | 0.05733 | no |  | 0.00112 | 0.00131 | ** |  |
| HNSC | 528 | 0.00347 | 0.00362 | ** |  | 0.00197 | 0.00212 | ** |  |
| KICH | 113 | 0.04090 | NA | * |  | 0.05826 | NA | no |  |
| KIRC | 537 | 0.11259 | 0.11335 | no |  | 0.05071 | 0.04955 | no |  |
| KIRP | 291 | 0.00007 | 0.00016 | *** |  | 0.00011 | 0.00024 | *** |  |
| LAML | 200 | 0.03045 | 0.03190 | * |  | NA | NA | NA |  |
| LGG | 515 | 0.00001 | 0.00001 | *** |  | 0.00009 | 0.00011 | *** |  |
| LIHC | 377 | 0.21565 | 0.21585 | no |  | 0.02287 | 0.02341 | * |  |
| LUAD | 522 | 0.00093 | 0.00110 | ** |  | 0.00174 | 0.00200 | ** |  |
| LUSC | 504 | 0.24097 | 0.23962 | no |  | 0.00494 | 0.00565 | ** |  |
| MESO | 87 | 0.00242 | 0.00301 | ** |  | 0.02653 | 0.02879 | * |  |
| OV | 587 | 0.05551 | 0.05721 | no |  | 0.03259 | 0.03333 | * |  |
| PAAD | 185 | 0.01198 | 0.01337 | * |  | 0.18357 | 0.18622 | no |  |
| PCPG | 179 | 0.06023 | NA | no |  | 0.09651 | 0.10496 | no |  |
| PRAD | 500 | 0.25253 | 0.26758 | no |  | 0.01236 | 0.01408 | * |  |
| READ | 170 | 0.03077 | 0.03488 | * |  | 0.20109 | 0.20364 | no |  |
| SARC | 261 | 0.00079 | 0.00097 | *** |  | 0.00200 | 0.00222 | ** |  |
| SKCM | 470 | 0.00001 | 0.00001 | *** |  | 0.00026 | 0.00028 | *** |  |
| STAD | 443 | 0.06136 | 0.06221 | no |  | 0.01404 | 0.01500 | * |  |
| TGCT | 134 | 0.00430 | 0.03046 | * |  | 0.08168 | 0.11647 | no |  |
| THCA | 507 | 0.09660 | 0.10547 | no |  | 0.03034 | 0.03440 | * |  |
| THYM | 124 | 0.00071 | 0.00574 | ** |  | 0.00785 | 0.01122 | ** |  |
| UCEC | 548 | 0.00090 | 0.00130 | *** |  | 0.04511 | 0.04613 | * |  |
| UCS | 57 | 0.38209 | 0.38503 | no |  | 0.13956 | 0.15164 | no |  |
| UVM | 80 | 0.00014 | 0.00056 | *** |  | 0.00630 | 0.01542 | ** |  |

TCGA, The Cancer Genome Atlas; N, number of patients; OS, overall survival; PFI, progression free interval; KM, Kaplan-Meier; CoxPH, Cox Proportional Hazards. NA: Not Available (no data or not sufficient data to calculate *p*-value). Significant: no, *p*-value > 0.05; *, 0.01 < *p*-value < 0.05; **, 0.001 < *p*-value < 0.01, ***, *p*-value < 0.001.

**Table S2. Results of survival analyses of 33 types of cancer from TCGA.**

|  |  | **Effect of *IDO1* on prognosis** | **OS** | | | |  | | **PFI** | | | | |  |
| --- | --- | --- | --- | --- | --- | --- | --- | --- | --- | --- | --- | --- | --- | --- |
| **TCGA code** | **N** |  | **Use^53^** | **Survival difference** | **HR** | **Significant** |  | **Use^53^** | | **Survival difference** | **HR** | **Significant** | | |
| ACC | 92 | NS | √ | -599 | 1.66 | no |  | √ | | 1827 | 0.16 | * |  |  |
| BLCA | 412 | Protective | √ | 688 | 0.63 | * |  | √ | | 568 | 0.71 | * |  |  |
| BRCA | 1097 | Protective | √^*^ | 1245 | 0.44 | ** |  | √ | | 1810 | 0.62 | ** |  |  |
| CESC | 307 | Protective | √ | 602 | 0.45 | * |  | √ | | 1710 | 0.21 | ** |  |  |
| CHOL | 45 | Protective | √ | 504 | 0.39 | * |  | √ | | 613 | 0.20 | *** |  |  |
| COAD | 459 | Protective | √ | 345 | 0.65 | * |  | √ | | 791 | 0.56 | ** |  |  |
| DLBC | 48 | NS | × |  |  |  |  | √^*^ | | -988 | 3.33 | no |  |  |
| ESCA | 185 | NS | √ | -527 | 1.98 | * |  | √ | | 373 | 0.67 | no |  |  |
| GBM | 596 | Deleterious | √ | -136 | 1.42 | no |  | √ | | -185 | 1.78 | ** |  |  |
| HNSC | 528 | Protective | √ | 694 | 0.69 | ** |  | √ | | 482 | 0.65 | ** |  |  |
| KICH | 113 | NS | √^*^ | -761 | NA | * |  | √^*^ | | -862 | NA | no |  |  |
| KIRC | 537 | NS | √ | -304 | 1.29 | no |  | √ | | 385 | 0.72 | no |  |  |
| KIRP | 291 | Deleterious | √ | -981 | 3.07 | *** |  | √ | | -846 | 3.16 | *** |  |  |
| LAML | 200 | Deleterious | √ | -327 | 1.55 | * |  | NA | | NA | NA | NA |  |  |
| LGG | 515 | Deleterious | √^*^ | -1089 | 2.46 | *** |  | √ | | -484 | 1.72 | *** |  |  |
| LIHC | 377 | Protective | √ | 140 | 0.82 | no |  | √ | | 282 | 0.71 | * |  |  |
| LUAD | 522 | Protective | √ | 895 | 0.55 | ** |  | √ | | 428 | 0.57 | ** |  |  |
| LUSC | 504 | NS | √ | -218 | 1.17 | no |  | √ | | 373 | 0.55 | ** |  |  |
| MESO | 87 | Protective | √ | 346 | 0.47 | ** |  | √ | | 275 | 0.55 | * |  |  |
| OV | 587 | Protective | √ | 309 | 0.67 | no |  | √ | | 288 | 0.74 | * |  |  |
| PAAD | 185 | Deleterious | √ | -431 | 1.83 | * |  | √ | | -225 | 1.30 | no |  |  |
| PCPG | 179 | NS | × |  |  |  |  | × | |  |  |  |  |  |
| PRAD | 500 | Deleterious | √^*^ | -294 | 0.41 | no |  | √ | | -615 | 1.96 | * |  |  |
| READ | 170 | NS | √^*^ | 1409 | 0.43 | * |  | √ | | 89 | 0.65 | no |  |  |
| SARC | 261 | Protective | √ | 866 | 0.51 | *** |  | √ | | 911 | 0.60 | ** |  |  |
| SKCM | 470 | Protective | √ | 2529 | 0.41 | *** |  | √ | | 916 | 0.66 | *** |  |  |
| STAD | 443 | NS | √ | 617 | 0.73 | no |  | √ | | -442 | 1.64 | * |  |  |
| TGCT | 134 | NS | × |  |  |  |  | √ | | 2223 | 0.20 | no |  |  |
| THCA | 507 | Deleterious | √^*^ | 180 | 0.45 | no |  | √ | | -414 | 2.14 | * |  |  |
| THYM | 124 | Deleterious | × |  |  |  |  | √ | | -854 | 3.06 | ** |  |  |
| UCEC | 548 | NS | √ | -1536 | 2.39 | *** |  | √ | | 420 | 0.69 | * |  |  |
| UCS | 57 | NS | √ | -454 | 1.53 | no |  | √ | | -1192 | 2.38 | no |  |  |
| UVM | 80 | Deleterious | √ | -772 | 5.45 | *** |  | √ | | -806 | 6.04 | ** |  |  |

√, recommended for use; X, not recommended for use; √^*^, caution. When an endpoint is not recommended for use, its corresponding cells are blank.

N, number of patients; OS, overall survival; PFI, progression free interval; HR, hazard ratio, the ratio of hazard rate in high and low *IDO1* groups; Survival difference, measured by mean survival time in days of *IDO1* high group minus mean survival time in days of *IDO1* low group. HR > 1 and survival difference < 0 indicate that *IDO1* high expression correlates to poor prognosis. HR < 1 and survival difference > 0 indicate that *IDO1* high expression correlates to good prognosis. Significant: no, *p*-value > 0.05; *, 0.01 < *p*-value < 0.05; **, 0.001 < *p*-value < 0.01; ***, p-value < 0.001.

**Table S3. Details of the candidate genes.**

| **Gene** | **Fold change** | **Co-expression** | **Gene ontology** | **KEGG pathway** | **Protein-Protein interaction** |
| --- | --- | --- | --- | --- | --- |
| ABCC8 | 1 | 0 | 1 | 0 | 0 |
| ACADL | 1 | 0 | 0 | 0 | 0 |
| ADA | 0 | 0 | 1 | 0 | 0 |
| ADAM6 | 1 | 0 | 0 | 0 | 0 |
| ADAM8 | 0 | 0 | 1 | 0 | 0 |
| ADAMTS2 | 0 | 0 | 0 | 0 | 1 |
| ADCY2 | 0 | 0 | 0 | 1 | 1 |
| ADCY5 | 0 | 0 | 0 | 1 | 1 |
| ADCY8 | 1 | 0 | 0 | 1 | 1 |
| ADCYAP1R1 | 0 | 0 | 0 | 0 | 1 |
| ADORA2B | 0 | 0 | 0 | 0 | 1 |
| ADRA1A | 0 | 0 | 0 | 0 | 1 |
| ADRB1 | 0 | 0 | 0 | 1 | 1 |
| ADRB2 | 0 | 0 | 0 | 1 | 0 |
| AGT | 0 | 0 | 0 | 0 | 1 |
| AIM1L | 1 | 0 | 0 | 0 | 0 |
| AKR1B10 | 1 | 0 | 0 | 0 | 0 |
| AKT3 | 0 | 0 | 0 | 1 | 0 |
| ALDH3B2 | 1 | 0 | 0 | 0 | 0 |
| ANLN | 0 | 0 | 0 | 0 | 1 |
| ANXA8 | 1 | 0 | 0 | 0 | 0 |
| AQP4 | 1 | 0 | 0 | 0 | 0 |
| ARG1 | 0 | 0 | 1 | 0 | 0 |
| ARHGAP11A | 0 | 1 | 0 | 0 | 1 |
| ARHGAP36 | 1 | 0 | 0 | 0 | 0 |
| ASF1B | 0 | 1 | 0 | 0 | 0 |
| ASPM | 1 | 1 | 0 | 0 | 1 |
| ATCAY | 1 | 0 | 0 | 0 | 0 |
| ATP1A2 | 1 | 0 | 0 | 0 | 0 |
| ATP1B2 | 1 | 0 | 0 | 0 | 0 |
| AURKA | 0 | 1 | 0 | 0 | 1 |
| AURKB | 0 | 1 | 0 | 0 | 1 |
| B3GAT1 | 1 | 0 | 0 | 0 | 0 |
| BAK1 | 0 | 0 | 0 | 1 | 0 |
| BCAN | 0 | 0 | 0 | 0 | 1 |
| BDKRB1 | 0 | 0 | 0 | 0 | 1 |
| BDKRB2 | 0 | 0 | 0 | 0 | 1 |
| BEX1 | 1 | 0 | 0 | 0 | 0 |
| BIRC5 | 0 | 1 | 0 | 0 | 1 |
| BST2 | 0 | 0 | 0 | 0 | 1 |
| BUB1 | 0 | 1 | 0 | 0 | 1 |
| BUB1B | 0 | 1 | 0 | 0 | 1 |
| C21orf62 | 1 | 0 | 0 | 0 | 0 |
| C2orf40 | 1 | 0 | 0 | 0 | 0 |
| C4BPA | 1 | 0 | 0 | 0 | 0 |
| CACNA1D | 0 | 0 | 0 | 1 | 0 |
| CALML5 | 1 | 0 | 0 | 0 | 0 |
| CAMK2A | 0 | 0 | 0 | 1 | 0 |
| CAMK2B | 1 | 0 | 0 | 1 | 1 |
| CASC5 | 0 | 0 | 0 | 0 | 1 |
| CASP14 | 0 | 0 | 0 | 0 | 1 |
| CASP3 | 0 | 0 | 0 | 1 | 0 |
| CASP8 | 0 | 0 | 0 | 1 | 0 |
| CAV1 | 0 | 0 | 1 | 0 | 0 |
| CBLC | 1 | 0 | 0 | 0 | 0 |
| CCK | 0 | 0 | 0 | 0 | 1 |
| CCKBR | 0 | 0 | 0 | 0 | 1 |
| CCL13 | 0 | 0 | 0 | 0 | 1 |
| CCL19 | 0 | 0 | 1 | 0 | 0 |
| CCL2 | 0 | 0 | 0 | 1 | 0 |
| CCL20 | 0 | 0 | 0 | 0 | 1 |
| CCL21 | 0 | 0 | 1 | 0 | 1 |
| CCL5 | 0 | 0 | 1 | 0 | 0 |
| CCNA2 | 0 | 1 | 0 | 1 | 1 |
| CCNB1 | 0 | 1 | 0 | 0 | 1 |
| CCNB2 | 0 | 1 | 0 | 0 | 1 |
| CCND1 | 0 | 0 | 0 | 1 | 0 |
| CCNE1 | 0 | 0 | 0 | 1 | 0 |
| CCNE2 | 0 | 0 | 0 | 1 | 0 |
| CCR7 | 0 | 0 | 1 | 0 | 0 |
| CD28 | 0 | 0 | 1 | 0 | 0 |
| CD36 | 0 | 0 | 1 | 0 | 0 |
| CD55 | 0 | 0 | 1 | 0 | 0 |
| CD80 | 0 | 0 | 1 | 0 | 0 |
| CDC20 | 0 | 1 | 0 | 0 | 1 |
| CDC25C | 0 | 1 | 0 | 0 | 1 |
| CDC45 | 0 | 1 | 0 | 0 | 1 |
| CDC6 | 0 | 1 | 0 | 0 | 1 |
| CDCA2 | 0 | 1 | 0 | 0 | 0 |
| CDCA3 | 0 | 0 | 0 | 0 | 1 |
| CDCA5 | 0 | 1 | 0 | 0 | 1 |
| CDCA8 | 0 | 1 | 0 | 0 | 1 |
| CDH10 | 1 | 0 | 0 | 0 | 0 |
| CDH2 | 0 | 0 | 0 | 0 | 1 |
| CDH22 | 1 | 0 | 0 | 0 | 0 |
| CDK1 | 0 | 1 | 0 | 0 | 1 |
| CDK2 | 0 | 0 | 0 | 1 | 0 |
| CDKN3 | 0 | 1 | 0 | 0 | 1 |
| CEACAM1 | 1 | 0 | 1 | 0 | 0 |
| CEACAM5 | 1 | 0 | 0 | 0 | 1 |
| CEACAM6 | 1 | 0 | 0 | 0 | 0 |
| CEACAM7 | 0 | 0 | 0 | 0 | 1 |
| CEBPB | 0 | 0 | 1 | 0 | 0 |
| CENPA | 0 | 1 | 0 | 0 | 1 |
| CENPE | 0 | 1 | 0 | 0 | 1 |
| CENPF | 0 | 0 | 0 | 0 | 1 |
| CENPI | 0 | 0 | 0 | 0 | 1 |
| CENPK | 0 | 0 | 0 | 0 | 1 |
| CENPM | 0 | 0 | 0 | 0 | 1 |
| CEP55 | 1 | 1 | 0 | 0 | 1 |
| CHD5 | 1 | 0 | 0 | 0 | 0 |
| CHGA | 1 | 0 | 0 | 0 | 0 |
| CHGB | 1 | 0 | 0 | 0 | 1 |
| CHRM1 | 0 | 0 | 0 | 0 | 1 |
| CHRNA2 | 1 | 0 | 0 | 0 | 0 |
| CKAP2L | 0 | 1 | 0 | 0 | 0 |
| CKS1B | 0 | 0 | 0 | 0 | 1 |
| CLDN10 | 1 | 0 | 0 | 0 | 0 |
| CNR1 | 0 | 0 | 0 | 0 | 1 |
| CNTN3 | 0 | 0 | 0 | 0 | 1 |
| CNTN4 | 0 | 0 | 0 | 0 | 1 |
| COL10A1 | 1 | 0 | 0 | 0 | 1 |
| COL11A1 | 0 | 0 | 0 | 0 | 1 |
| COL12A1 | 1 | 0 | 0 | 0 | 1 |
| COL15A1 | 0 | 0 | 0 | 0 | 1 |
| COL1A1 | 1 | 1 | 0 | 0 | 1 |
| COL1A2 | 1 | 1 | 0 | 0 | 1 |
| COL20A1 | 0 | 0 | 0 | 0 | 1 |
| COL23A1 | 0 | 0 | 0 | 0 | 1 |
| COL26A1 | 0 | 0 | 0 | 0 | 1 |
| COL3A1 | 1 | 1 | 0 | 0 | 1 |
| COL4A1 | 0 | 0 | 0 | 0 | 1 |
| COL4A3 | 1 | 0 | 0 | 0 | 1 |
| COL4A4 | 0 | 0 | 0 | 0 | 1 |
| COL5A1 | 1 | 1 | 0 | 0 | 1 |
| COL5A2 | 0 | 1 | 0 | 0 | 1 |
| COL5A3 | 0 | 0 | 0 | 0 | 1 |
| COL6A2 | 0 | 0 | 0 | 0 | 1 |
| COL6A3 | 1 | 1 | 0 | 0 | 1 |
| COL6A6 | 0 | 0 | 0 | 0 | 1 |
| COL9A1 | 0 | 0 | 0 | 0 | 1 |
| COLGALT2 | 0 | 0 | 0 | 0 | 1 |
| CPNE4 | 1 | 0 | 0 | 0 | 0 |
| CRHR1 | 0 | 0 | 0 | 0 | 1 |
| CSTA | 0 | 0 | 0 | 0 | 1 |
| CTHRC1 | 1 | 0 | 0 | 0 | 0 |
| CTNND2 | 1 | 0 | 0 | 0 | 0 |
| CX3CR1 | 0 | 0 | 0 | 0 | 1 |
| CXCL1 | 0 | 0 | 0 | 0 | 1 |
| CXCL10 | 1 | 0 | 0 | 1 | 1 |
| CXCL11 | 0 | 0 | 0 | 0 | 1 |
| CXCL13 | 1 | 0 | 0 | 0 | 1 |
| CXCL9 | 0 | 0 | 0 | 0 | 1 |
| CYSLTR2 | 0 | 0 | 0 | 0 | 1 |
| DBC1 | 1 | 0 | 0 | 0 | 0 |
| DCN | 0 | 0 | 0 | 0 | 1 |
| DEPDC1 | 0 | 1 | 0 | 0 | 1 |
| DIRAS2 | 1 | 0 | 0 | 0 | 0 |
| DLG2 | 1 | 0 | 0 | 0 | 1 |
| DLG4 | 0 | 0 | 0 | 0 | 1 |
| DLGAP5 | 1 | 1 | 0 | 0 | 1 |
| DMRTC1B | 1 | 0 | 0 | 0 | 0 |
| DNER | 1 | 0 | 0 | 0 | 0 |
| DPP6 | 1 | 0 | 0 | 0 | 0 |
| DSC3 | 0 | 0 | 0 | 0 | 1 |
| DSG1 | 0 | 0 | 0 | 0 | 1 |
| DSG2 | 0 | 0 | 0 | 0 | 1 |
| DSG3 | 1 | 0 | 0 | 0 | 1 |
| DSP | 0 | 0 | 0 | 0 | 1 |
| DTL | 0 | 0 | 0 | 0 | 1 |
| E2F1 | 0 | 0 | 0 | 1 | 0 |
| E2F2 | 0 | 0 | 0 | 1 | 0 |
| E2F8 | 0 | 1 | 0 | 0 | 0 |
| ECT2 | 0 | 0 | 0 | 0 | 1 |
| EDN3 | 1 | 0 | 0 | 0 | 1 |
| EGFL6 | 1 | 0 | 0 | 0 | 0 |
| EIF2AK2 | 0 | 0 | 0 | 1 | 0 |
| ELAVL3 | 1 | 0 | 0 | 0 | 0 |
| ELFN2 | 1 | 0 | 0 | 0 | 0 |
| ERCC6L | 0 | 1 | 0 | 0 | 1 |
| ESPL1 | 0 | 1 | 0 | 0 | 1 |
| EXO1 | 0 | 1 | 0 | 0 | 1 |
| F2RL2 | 0 | 0 | 0 | 0 | 1 |
| FADD | 0 | 0 | 1 | 1 | 0 |
| FAM107A | 1 | 0 | 0 | 0 | 0 |
| FAM181B | 1 | 0 | 0 | 0 | 0 |
| FAM54A | 0 | 1 | 0 | 0 | 0 |
| FAM5C | 1 | 0 | 0 | 0 | 0 |
| FAM83A | 1 | 0 | 0 | 0 | 0 |
| FANCI | 0 | 1 | 0 | 0 | 0 |
| FAP | 0 | 1 | 0 | 0 | 0 |
| FASLG | 0 | 0 | 0 | 1 | 0 |
| FCGR2B | 0 | 0 | 1 | 0 | 0 |
| FCGR3A | 0 | 0 | 0 | 1 | 0 |
| FCGR3B | 0 | 0 | 0 | 1 | 0 |
| FGA | 0 | 0 | 0 | 0 | 1 |
| FGB | 0 | 0 | 0 | 0 | 1 |
| FGG | 0 | 0 | 0 | 0 | 1 |
| FLJ16779 | 1 | 0 | 0 | 0 | 0 |
| FMN2 | 1 | 0 | 0 | 0 | 0 |
| FN1 | 0 | 0 | 0 | 0 | 1 |
| FOXA1 | 1 | 0 | 0 | 0 | 0 |
| FOXM1 | 0 | 1 | 0 | 0 | 1 |
| FOXP3 | 0 | 0 | 1 | 0 | 0 |
| FPR3 | 0 | 0 | 0 | 0 | 1 |
| FRK | 0 | 0 | 0 | 0 | 1 |
| FST | 1 | 0 | 0 | 0 | 0 |
| FXYD3 | 1 | 0 | 0 | 0 | 0 |
| GABBR2 | 1 | 0 | 0 | 0 | 1 |
| GABRB3 | 1 | 0 | 0 | 0 | 0 |
| GAL3ST3 | 1 | 0 | 0 | 0 | 0 |
| GALNT9 | 1 | 0 | 0 | 0 | 0 |
| GALR1 | 0 | 0 | 0 | 0 | 1 |
| GAP43 | 1 | 0 | 0 | 0 | 0 |
| GATA3 | 0 | 0 | 1 | 0 | 0 |
| GFAP | 1 | 0 | 0 | 0 | 0 |
| GGH | 0 | 0 | 0 | 0 | 1 |
| GJB2 | 1 | 0 | 0 | 0 | 0 |
| GLI3 | 0 | 0 | 1 | 0 | 0 |
| GNAQ | 0 | 0 | 0 | 1 | 0 |
| GNG3 | 0 | 0 | 0 | 0 | 1 |
| GNG7 | 0 | 0 | 0 | 0 | 1 |
| GPC3 | 0 | 0 | 0 | 0 | 1 |
| GPC5 | 0 | 0 | 0 | 0 | 1 |
| GPM6A | 1 | 0 | 0 | 0 | 0 |
| GPR17 | 0 | 0 | 0 | 0 | 1 |
| GPR37L1 | 0 | 0 | 0 | 0 | 1 |
| GPR68 | 0 | 0 | 0 | 0 | 1 |
| GPX2 | 1 | 0 | 0 | 0 | 0 |
| GREM1 | 0 | 0 | 1 | 0 | 0 |
| GRIA1 | 0 | 0 | 0 | 0 | 1 |
| GRIA2 | 0 | 0 | 0 | 0 | 1 |
| GRIK5 | 1 | 0 | 0 | 0 | 0 |
| GRIN1 | 0 | 0 | 1 | 0 | 1 |
| GRIN2A | 0 | 0 | 0 | 0 | 1 |
| GRIN2C | 0 | 0 | 0 | 0 | 1 |
| GRM3 | 0 | 0 | 0 | 0 | 1 |
| GRM5 | 0 | 0 | 0 | 0 | 1 |
| GSG2 | 0 | 1 | 0 | 0 | 0 |
| GTSE1 | 0 | 1 | 0 | 0 | 0 |
| H19 | 1 | 0 | 0 | 0 | 0 |
| HBB | 0 | 0 | 0 | 0 | 1 |
| HCAR2 | 0 | 0 | 0 | 0 | 1 |
| HCAR3 | 0 | 0 | 0 | 0 | 1 |
| HHATL | 1 | 0 | 0 | 0 | 0 |
| HJURP | 0 | 1 | 0 | 0 | 1 |
| HLA-B | 0 | 0 | 0 | 1 | 0 |
| HLA-C | 0 | 0 | 0 | 1 | 0 |
| HLA-DOB | 0 | 0 | 0 | 1 | 0 |
| HLA-DQA1 | 0 | 0 | 0 | 1 | 0 |
| HLA-DQA2 | 0 | 0 | 0 | 1 | 0 |
| HLA-DQB1 | 0 | 0 | 0 | 1 | 0 |
| HLA-DRA | 0 | 0 | 0 | 1 | 0 |
| HLA-DRB1 | 0 | 0 | 0 | 1 | 0 |
| HLA-DRB5 | 0 | 0 | 0 | 1 | 0 |
| HLA-F | 0 | 0 | 0 | 1 | 0 |
| HMGA2 | 0 | 0 | 1 | 0 | 0 |
| HMMR | 0 | 0 | 0 | 0 | 1 |
| HOXC13 | 1 | 0 | 0 | 0 | 0 |
| HRH3 | 0 | 0 | 0 | 0 | 1 |
| ICAM1 | 0 | 0 | 1 | 1 | 0 |
| IFNG | 0 | 0 | 1 | 1 | 0 |
| IGF2 | 0 | 0 | 0 | 0 | 1 |
| IGFBP3 | 0 | 0 | 0 | 0 | 1 |
| IGSF1 | 1 | 0 | 0 | 0 | 0 |
| IL10 | 0 | 0 | 1 | 0 | 0 |
| IL12B | 0 | 0 | 1 | 0 | 0 |
| IL1A | 0 | 0 | 0 | 1 | 0 |
| IL1B | 0 | 0 | 1 | 1 | 0 |
| IL1RN | 1 | 0 | 0 | 0 | 0 |
| IL23A | 0 | 0 | 1 | 0 | 0 |
| IL2RA | 0 | 0 | 0 | 1 | 0 |
| IL2RB | 0 | 0 | 0 | 1 | 0 |
| IL2RG | 0 | 0 | 0 | 1 | 0 |
| IL6 | 0 | 0 | 1 | 1 | 0 |
| IRF1 | 0 | 0 | 1 | 0 | 0 |
| IRF6 | 0 | 0 | 0 | 0 | 1 |
| IRF7 | 0 | 0 | 0 | 1 | 0 |
| ITGA2 | 0 | 0 | 0 | 1 | 0 |
| ITGA5 | 0 | 0 | 0 | 1 | 0 |
| ITGB1 | 0 | 0 | 0 | 1 | 0 |
| ITGB5 | 0 | 0 | 0 | 1 | 0 |
| ITPR1 | 0 | 0 | 0 | 1 | 0 |
| IVL | 0 | 0 | 0 | 0 | 1 |
| KCNAB1 | 0 | 0 | 0 | 0 | 1 |
| KCNJ16 | 1 | 0 | 0 | 0 | 0 |
| KCNK3 | 1 | 0 | 0 | 0 | 0 |
| KCNQ2 | 1 | 0 | 0 | 0 | 0 |
| KCTD8 | 1 | 0 | 0 | 0 | 0 |
| KDELR3 | 0 | 0 | 0 | 0 | 1 |
| KIAA0101 | 0 | 0 | 0 | 0 | 1 |
| KIF11 | 0 | 1 | 0 | 0 | 1 |
| KIF14 | 0 | 1 | 0 | 0 | 0 |
| KIF15 | 0 | 0 | 0 | 0 | 1 |
| KIF18A | 0 | 0 | 0 | 0 | 1 |
| KIF18B | 0 | 0 | 0 | 0 | 1 |
| KIF1A | 1 | 0 | 0 | 0 | 1 |
| KIF20A | 0 | 1 | 0 | 0 | 1 |
| KIF23 | 0 | 1 | 0 | 0 | 1 |
| KIF2C | 0 | 1 | 0 | 0 | 1 |
| KIF4A | 0 | 1 | 0 | 0 | 1 |
| KIF4B | 0 | 0 | 0 | 0 | 1 |
| KIF5A | 0 | 0 | 0 | 0 | 1 |
| KIFC1 | 0 | 1 | 0 | 0 | 1 |
| KLK2 | 1 | 0 | 0 | 0 | 0 |
| KLK3 | 1 | 0 | 0 | 0 | 0 |
| KNDC1 | 1 | 0 | 0 | 0 | 0 |
| KRT1 | 0 | 0 | 0 | 0 | 1 |
| KRT13 | 0 | 0 | 0 | 0 | 1 |
| KRT14 | 1 | 0 | 0 | 0 | 1 |
| KRT15 | 0 | 0 | 0 | 0 | 1 |
| KRT16 | 1 | 1 | 0 | 0 | 1 |
| KRT17 | 1 | 0 | 0 | 0 | 1 |
| KRT18 | 0 | 0 | 0 | 0 | 1 |
| KRT19 | 0 | 0 | 0 | 0 | 1 |
| KRT23 | 1 | 0 | 0 | 0 | 1 |
| KRT4 | 0 | 0 | 0 | 0 | 1 |
| KRT5 | 1 | 0 | 0 | 0 | 1 |
| KRT6A | 1 | 1 | 0 | 0 | 1 |
| KRT6B | 1 | 0 | 0 | 0 | 1 |
| KRT6C | 1 | 1 | 0 | 0 | 1 |
| KRT75 | 0 | 0 | 0 | 0 | 1 |
| KRT8 | 0 | 0 | 0 | 0 | 1 |
| KRT80 | 0 | 0 | 0 | 0 | 1 |
| LAD1 | 1 | 0 | 0 | 0 | 0 |
| LAMA3 | 0 | 0 | 0 | 0 | 1 |
| LAMB3 | 0 | 0 | 0 | 0 | 1 |
| LAMC2 | 0 | 0 | 0 | 0 | 1 |
| LEP | 0 | 0 | 1 | 0 | 0 |
| LGALS9 | 0 | 0 | 1 | 0 | 0 |
| LGI3 | 1 | 0 | 0 | 0 | 0 |
| LHFPL4 | 1 | 0 | 0 | 0 | 0 |
| LIX1 | 1 | 0 | 0 | 0 | 0 |
| LMO3 | 1 | 0 | 0 | 0 | 0 |
| LOC286002 | 1 | 0 | 0 | 0 | 0 |
| LOC643763 | 1 | 0 | 0 | 0 | 0 |
| LONRF2 | 1 | 0 | 0 | 0 | 0 |
| LRG1 | 0 | 0 | 0 | 0 | 1 |
| LRRC15 | 1 | 0 | 0 | 0 | 0 |
| LUM | 1 | 0 | 0 | 0 | 1 |
| LY6D | 0 | 0 | 0 | 0 | 1 |
| LY6K | 0 | 0 | 0 | 0 | 1 |
| LYPD3 | 1 | 0 | 0 | 0 | 1 |
| LYPD6B | 0 | 0 | 0 | 0 | 1 |
| MAG | 1 | 0 | 0 | 0 | 0 |
| MAPK13 | 0 | 0 | 0 | 1 | 0 |
| MAPK4 | 1 | 0 | 0 | 0 | 0 |
| MCM10 | 0 | 1 | 0 | 0 | 1 |
| MELK | 0 | 1 | 0 | 0 | 1 |
| MFAP5 | 1 | 0 | 0 | 0 | 0 |
| MGAT4C | 1 | 0 | 0 | 0 | 0 |
| MIF | 0 | 0 | 1 | 0 | 0 |
| MKI67 | 0 | 1 | 0 | 0 | 1 |
| MLC1 | 1 | 0 | 0 | 0 | 0 |
| MMP1 | 1 | 0 | 0 | 0 | 1 |
| MMP11 | 1 | 0 | 0 | 0 | 0 |
| MMP12 | 1 | 0 | 0 | 0 | 0 |
| MMP13 | 1 | 0 | 0 | 0 | 1 |
| MMP14 | 0 | 0 | 1 | 0 | 0 |
| MMP2 | 0 | 0 | 0 | 0 | 1 |
| MMP3 | 1 | 0 | 0 | 0 | 1 |
| MMP9 | 0 | 0 | 0 | 0 | 1 |
| MOCOS | 1 | 0 | 0 | 0 | 0 |
| MT3 | 1 | 0 | 0 | 0 | 0 |
| MUC1 | 0 | 0 | 0 | 0 | 1 |
| MUC16 | 0 | 0 | 0 | 0 | 1 |
| MUC5B | 1 | 0 | 0 | 0 | 1 |
| MUCL1 | 0 | 0 | 0 | 0 | 1 |
| MYBL2 | 1 | 1 | 0 | 0 | 0 |
| MYC | 0 | 0 | 0 | 1 | 0 |
| MYD88 | 0 | 0 | 0 | 1 | 0 |
| MYEOV | 1 | 0 | 0 | 0 | 0 |
| NAP1L2 | 1 | 0 | 0 | 0 | 0 |
| NCAM1 | 0 | 0 | 0 | 0 | 1 |
| NCAN | 1 | 0 | 0 | 0 | 1 |
| NCAPG | 0 | 1 | 0 | 0 | 1 |
| NCAPH | 0 | 1 | 0 | 0 | 1 |
| NDC80 | 0 | 1 | 0 | 0 | 1 |
| NEFL | 0 | 0 | 0 | 0 | 1 |
| NEK2 | 1 | 1 | 0 | 0 | 1 |
| NKAIN4 | 1 | 0 | 0 | 0 | 0 |
| NLGN1 | 0 | 0 | 1 | 0 | 0 |
| NME5 | 1 | 0 | 0 | 0 | 0 |
| NOD2 | 0 | 0 | 1 | 0 | 0 |
| NOL4 | 1 | 0 | 0 | 0 | 0 |
| NOTUM | 0 | 0 | 0 | 0 | 1 |
| NOVA1 | 1 | 0 | 0 | 0 | 0 |
| NPY | 1 | 0 | 0 | 0 | 1 |
| NRAS | 0 | 0 | 0 | 1 | 0 |
| NRCAM | 1 | 0 | 0 | 0 | 0 |
| NRSN1 | 1 | 0 | 0 | 0 | 0 |
| NRXN1 | 0 | 0 | 0 | 0 | 1 |
| NRXN2 | 0 | 0 | 0 | 0 | 1 |
| NTNG1 | 0 | 0 | 0 | 0 | 1 |
| NTNG2 | 0 | 0 | 0 | 0 | 1 |
| NTRK2 | 1 | 0 | 0 | 0 | 0 |
| NTRK3 | 1 | 0 | 0 | 0 | 0 |
| NTSR2 | 0 | 0 | 0 | 0 | 1 |
| NUF2 | 0 | 1 | 0 | 0 | 1 |
| NUSAP1 | 0 | 1 | 0 | 0 | 1 |
| OAS1 | 0 | 0 | 0 | 1 | 1 |
| OAS2 | 0 | 0 | 0 | 1 | 0 |
| OAS3 | 0 | 0 | 0 | 1 | 0 |
| OASL | 0 | 0 | 0 | 0 | 1 |
| ODZ1 | 1 | 0 | 0 | 0 | 0 |
| OGDHL | 1 | 0 | 0 | 0 | 0 |
| OIP5 | 0 | 1 | 0 | 0 | 1 |
| OLIG1 | 1 | 0 | 0 | 0 | 0 |
| OLIG2 | 1 | 0 | 0 | 0 | 0 |
| OPCML | 0 | 0 | 0 | 0 | 1 |
| OPRK1 | 0 | 0 | 0 | 0 | 1 |
| ORC1L | 0 | 1 | 0 | 0 | 0 |
| P2RY12 | 0 | 0 | 0 | 0 | 1 |
| P2RY2 | 0 | 0 | 0 | 0 | 1 |
| P4HA2 | 0 | 0 | 0 | 0 | 1 |
| PBK | 0 | 0 | 0 | 0 | 1 |
| PCDH10 | 1 | 0 | 0 | 0 | 0 |
| PCDH8 | 1 | 0 | 0 | 0 | 0 |
| PCOLCE | 0 | 0 | 0 | 0 | 1 |
| PCP4 | 1 | 0 | 0 | 0 | 0 |
| PCSK1N | 1 | 0 | 0 | 0 | 0 |
| PCSK2 | 1 | 0 | 0 | 0 | 0 |
| PCSK9 | 1 | 0 | 0 | 0 | 1 |
| PDE8B | 1 | 0 | 0 | 0 | 0 |
| PDGFRB | 0 | 0 | 0 | 1 | 0 |
| PEBP4 | 1 | 0 | 0 | 0 | 0 |
| PHF21B | 1 | 0 | 0 | 0 | 0 |
| PHYHIPL | 1 | 0 | 0 | 0 | 0 |
| PI3 | 1 | 0 | 0 | 0 | 1 |
| PIK3R3 | 0 | 0 | 0 | 1 | 0 |
| PITX1 | 1 | 0 | 0 | 0 | 0 |
| PITX2 | 1 | 0 | 0 | 0 | 0 |
| PKP2 | 0 | 0 | 0 | 0 | 1 |
| PKP3 | 0 | 0 | 0 | 0 | 1 |
| PLAU | 0 | 0 | 0 | 0 | 1 |
| PLAUR | 0 | 0 | 0 | 0 | 1 |
| PLCB1 | 0 | 0 | 0 | 1 | 0 |
| PLCB4 | 0 | 0 | 0 | 1 | 0 |
| PLK1 | 0 | 1 | 0 | 0 | 1 |
| PLK4 | 0 | 1 | 0 | 0 | 0 |
| PLP1 | 1 | 0 | 0 | 0 | 0 |
| PMP2 | 1 | 0 | 0 | 0 | 0 |
| POF1B | 1 | 0 | 0 | 0 | 0 |
| POLE2 | 0 | 0 | 0 | 0 | 1 |
| POLQ | 0 | 1 | 0 | 0 | 0 |
| POSTN | 1 | 0 | 0 | 0 | 0 |
| POU3F3 | 1 | 0 | 0 | 0 | 0 |
| PPARG | 0 | 0 | 1 | 0 | 0 |
| PPP3CA | 0 | 0 | 0 | 1 | 0 |
| PRC1 | 0 | 1 | 0 | 0 | 1 |
| PRKACB | 0 | 0 | 0 | 1 | 0 |
| PRKCA | 0 | 0 | 0 | 1 | 0 |
| PROK1 | 0 | 0 | 0 | 0 | 1 |
| PTHLH | 0 | 0 | 0 | 0 | 1 |
| PTK2B | 0 | 0 | 1 | 0 | 0 |
| PTPN22 | 0 | 0 | 1 | 0 | 0 |
| PTPRN2 | 1 | 0 | 0 | 0 | 0 |
| PTTG1 | 0 | 0 | 0 | 0 | 1 |
| PVRL4 | 1 | 0 | 0 | 0 | 0 |
| PYCARD | 0 | 0 | 1 | 0 | 0 |
| RACGAP1 | 0 | 0 | 0 | 0 | 1 |
| RAD51 | 0 | 1 | 0 | 0 | 1 |
| RAD54L | 0 | 1 | 0 | 0 | 0 |
| RFX4 | 1 | 0 | 0 | 0 | 0 |
| RHOD | 0 | 0 | 0 | 0 | 1 |
| RHOV | 0 | 0 | 0 | 0 | 1 |
| RIC3 | 1 | 0 | 0 | 0 | 0 |
| RMST | 1 | 0 | 0 | 0 | 0 |
| RRM2 | 0 | 1 | 0 | 0 | 1 |
| S100A14 | 1 | 0 | 0 | 0 | 0 |
| S100A7 | 1 | 0 | 0 | 0 | 0 |
| S100A9 | 1 | 0 | 0 | 0 | 0 |
| S100P | 1 | 0 | 0 | 0 | 0 |
| SAA1 | 1 | 0 | 0 | 0 | 1 |
| SALL1 | 1 | 0 | 0 | 0 | 0 |
| SALL3 | 1 | 0 | 0 | 0 | 0 |
| SCG3 | 1 | 0 | 0 | 0 | 1 |
| SCRG1 | 1 | 0 | 0 | 0 | 0 |
| SDC1 | 0 | 0 | 0 | 0 | 1 |
| SDR16C5 | 1 | 0 | 0 | 0 | 0 |
| SERPINB5 | 1 | 0 | 0 | 0 | 0 |
| SERPINE1 | 0 | 0 | 1 | 0 | 1 |
| SFN | 1 | 0 | 0 | 0 | 0 |
| SFRP2 | 0 | 0 | 1 | 0 | 0 |
| SGOL1 | 0 | 1 | 0 | 0 | 1 |
| SH3GL2 | 1 | 0 | 0 | 0 | 1 |
| SHCBP1 | 0 | 1 | 0 | 0 | 0 |
| SKA1 | 0 | 1 | 0 | 0 | 1 |
| SKA3 | 0 | 1 | 0 | 0 | 0 |
| SLAIN1 | 1 | 0 | 0 | 0 | 0 |
| SLC4A4 | 1 | 0 | 0 | 0 | 0 |
| SLC6A13 | 1 | 0 | 0 | 0 | 0 |
| SLIT1 | 1 | 0 | 0 | 0 | 0 |
| SLITRK5 | 1 | 0 | 0 | 0 | 0 |
| SLITRK6 | 1 | 0 | 0 | 0 | 0 |
| SNAP25 | 0 | 0 | 0 | 0 | 1 |
| SNAP91 | 1 | 0 | 0 | 0 | 0 |
| SOX8 | 1 | 0 | 0 | 0 | 0 |
| SPAG5 | 0 | 0 | 0 | 0 | 1 |
| SPC24 | 0 | 0 | 0 | 0 | 1 |
| SPC25 | 0 | 0 | 0 | 0 | 1 |
| SPOCK3 | 1 | 0 | 0 | 0 | 0 |
| SPRR1A | 0 | 1 | 0 | 0 | 1 |
| SPRR1B | 0 | 1 | 0 | 0 | 1 |
| SPRR2A | 0 | 0 | 0 | 0 | 1 |
| SPRR2D | 0 | 0 | 0 | 0 | 1 |
| SPRR2E | 0 | 0 | 0 | 0 | 1 |
| SPRR2F | 0 | 0 | 0 | 0 | 1 |
| SPRR3 | 0 | 0 | 0 | 0 | 1 |
| SST | 0 | 0 | 0 | 0 | 1 |
| SSTR1 | 0 | 0 | 0 | 0 | 1 |
| STAT1 | 0 | 0 | 0 | 1 | 0 |
| STC2 | 0 | 0 | 0 | 0 | 1 |
| STK33 | 1 | 0 | 0 | 0 | 0 |
| STXBP5L | 1 | 0 | 0 | 0 | 0 |
| SYT4 | 1 | 0 | 0 | 0 | 0 |
| TAC1 | 0 | 0 | 0 | 0 | 1 |
| TACC3 | 0 | 0 | 0 | 0 | 1 |
| TAGLN3 | 1 | 0 | 0 | 0 | 0 |
| TCEAL2 | 1 | 0 | 0 | 0 | 0 |
| TCERG1L | 1 | 0 | 0 | 0 | 0 |
| TCN1 | 0 | 0 | 0 | 0 | 1 |
| TG | 1 | 0 | 0 | 0 | 0 |
| THBS1 | 0 | 0 | 1 | 1 | 1 |
| TMEFF2 | 1 | 0 | 0 | 0 | 0 |
| TMEM59L | 1 | 0 | 0 | 0 | 0 |
| TNF | 0 | 0 | 1 | 1 | 0 |
| TNFAIP3 | 0 | 0 | 1 | 0 | 0 |
| TNFRSF10A | 0 | 0 | 0 | 1 | 0 |
| TNFRSF1A | 0 | 0 | 0 | 1 | 0 |
| TNFSF10 | 0 | 0 | 0 | 1 | 0 |
| TNNT1 | 1 | 0 | 0 | 0 | 0 |
| TOP2A | 0 | 1 | 0 | 0 | 1 |
| TP63 | 0 | 0 | 1 | 0 | 0 |
| TPX2 | 0 | 1 | 0 | 0 | 1 |
| TRIP13 | 0 | 1 | 0 | 0 | 0 |
| TROAP | 0 | 1 | 0 | 0 | 0 |
| TSHR | 1 | 0 | 0 | 0 | 1 |
| TTK | 0 | 1 | 0 | 0 | 1 |
| UBE2C | 1 | 1 | 0 | 0 | 1 |
| UNC5D | 1 | 0 | 0 | 0 | 0 |
| UNC80 | 1 | 0 | 0 | 0 | 0 |
| VIPR2 | 0 | 0 | 0 | 0 | 1 |
| WNT3A | 0 | 0 | 1 | 0 | 0 |
| WNT4 | 0 | 0 | 1 | 0 | 0 |
| XBP1 | 0 | 0 | 1 | 0 | 0 |
| XCL1 | 0 | 0 | 1 | 0 | 0 |
| ZBTB16 | 1 | 0 | 0 | 0 | 0 |
| ZC3H12A | 0 | 0 | 1 | 0 | 0 |
| ZWINT | 0 | 0 | 0 | 0 | 1 |
| 1/0: This gene is/is not identified in the specified procedure. | | | |  |  |

**Table S4. 43 key genes associated with the effect of *IDO1* on prognosis.**

| **Gene** | **Frequency** | **Protein** | **IDO1 risk** |
| --- | --- | --- | --- |
| GRIA1 | 22 | glutamate ionotropic receptor AMPA type subunit 1 | + |
| FCGR3A | 19 | Fc fragment of IgG receptor IIIa | + |
| GSG2 | 16 | histone H3 associated protein kinase | - |
| GNG3 | 12 | G protein subunit gamma 3 | + |
| GRIA2 | 12 | glutamate ionotropic receptor AMPA type subunit 2 | + |
| IVL | 12 | involucrin | - |
| CALML5 | 11 | calmodulin like 5 | - |
| LY6D | 11 | lymphocyte antigen 6 family member D | - |
| RMST | 11 | rhabdomyosarcoma 2 associated transcript | + |
| CCL19 | 10 | C-C motif chemokine ligand 19 | - |
| GPX2 | 10 | glutathione peroxidase 2 | - |
| XCL1 | 10 | X-C motif chemokine ligand 1 | - |
| BST2 | 9 | bone marrow stromal cell antigen 2 | - |
| CKS1B | 9 | CDC28 protein kinase regulatory subunit 1B | - |
| NRCAM | 9 | neuronal cell adhesion molecule | + |
| NTNG2 | 9 | netrin G2 | + |
| OPCML | 9 | opioid binding protein/cell adhesion molecule like | + |
| TACC3 | 9 | transforming acidic coiled-coil containing protein 3 | - |
| ALDH3B2 | 8 | aldehyde dehydrogenase 3 family member B2 | - |
| CENPA | 8 | centromere protein A | - |
| GGH | 8 | gamma-glutamyl hydrolase | - |
| GPM6A | 8 | glycoprotein M6A | + |
| MMP1 | 8 | matrix metallopeptidase 1 | - |
| P2RY12 | 8 | purinergic receptor P2Y12 | + |
| SERPINB5 | 8 | serpin family B member 5 | - |
| CAMK2B | 7 | calcium/calmodulin dependent protein kinase II beta | + |
| CCL21 | 7 | C-C motif chemokine ligand 21 | - |
| FOXM1 | 7 | forkhead box M1 | - |
| GFAP | 7 | glial fibrillary acidic protein | + |
| GTSE1 | 7 | G2 and S-phase expressed 1 | - |
| IL12B | 7 | interleukin 12B | - |
| KRT4 | 7 | keratin 4 | - |
| LONRF2 | 7 | LON peptidase N-terminal domain and ring finger 2 | + |
| PTPRN2 | 7 | protein tyrosine phosphatase receptor type N2 | + |
| PVRL4 | 7 | nectin cell adhesion molecule 4 | - |
| CCR7 | 6 | C-C motif chemokine receptor 7 | - |
| DPP6 | 6 | dipeptidyl peptidase like 6 | + |
| KRT13 | 6 | keratin 13 | - |
| KRT14 | 6 | keratin 14 | - |
| KRT18 | 6 | keratin 18 | + |
| MMP14 | 6 | matrix metallopeptidase 14 | - |
| NME5 | 6 | NME/NM23 family member 5 | + |
| PCSK2 | 6 | proprotein convertase subtilisin/kexin type 2 | - |

Frequency: the number of times a variable was selected for splitting. IDO1 risk: +, *IDO1* high expression correlated to poor prognosis in patients with high expression of the corresponding gene; -, *IDO1* high expression correlated to good prognosis in patients with high expression of the corresponding gene. The frequency of patient category is 7.

**Table S5. Interpretation of new key genes (refer to Figure S9).**

| **Gene** | **R-squared** | **Significant** |
| --- | --- | --- |
| ANLN | 0.831274148 | *** |
| APOL1 | 0.633599099 | *** |
| CCL13 | 0.580870212 | *** |
| CCL20 | 0.527758235 | *** |
| CCNE1 | 0.695333273 | *** |
| CDC6 | 0.862255687 | *** |
| CDCA8 | 0.94130611 | *** |
| CDH10 | 0.659222213 | *** |
| CDH1 | 0.672200497 | *** |
| COL23A1 | 0.458281006 | *** |
| ESPL1 | 0.867447655 | *** |
| FAM83A | 0.71556434 | *** |
| FANCI | 0.861459375 | *** |
| GATA3 | 0.503703809 | *** |
| GPC5 | 0.470836524 | *** |
| GPR37L1 | 0.686336408 | *** |
| GRIN1 | 0.566425029 | *** |
| GRM5 | 0.502981762 | *** |
| HLA.F | 0.55413568 | *** |
| ICAM1 | 0.598773659 | *** |
| IL23A | 0.48209113 | *** |
| KIF18B | 0.925699384 | *** |
| KIFC1 | 0.935611777 | *** |
| KRT5 | 0.866910786 | *** |
| KRT6A | 0.862202301 | *** |
| KRT6C | 0.862735471 | *** |
| MMP9 | 0.619464984 | *** |
| MYBL2 | 0.921179191 | *** |
| ORC1L | 0.885015658 | *** |
| PTTG1 | 0.795588991 | *** |
| S100A9 | 0.736433174 | *** |
| SFRP2 | 0.583251984 | *** |
| SPAG5 | 0.771135376 | *** |
| STAT2 | 0.44258401 | *** |
| SYT9 | 0.502451811 | *** |
| TNFAIP3 | 0.527770917 | *** |

We randomly selected 4408 patients from IDO1 protective and deleterious categories as the new discovery set, performed the same procedure as described in the text, and then got 46 new key genes. We fitted the expression of each new key gene with the expression of 43 key genes described in Table S5 by linear regression. R-squareds indicate how much variation of a new key gene is explained by the 43 key genes in the linear regression models. The last two columns showed that expression of 43 key genes, which we listed in Table S5, can explain the expression of new key genes well. ***, *p*-value < 0.001.

**Table S6. EGFR inhibitors could still selectively eradicate cell lines with the high new 46-gene score (refer to Figure S9).**

| **Drug name** | **Drug ID** | **Coefficient** | **P-value** |
| --- | --- | --- | --- |
| Afatinib | 1032 | 0.62 | 0.0075 |
| Afatinib | 1377 | 0.43 | 0.059 |
| Cetuximab | 1114 | 0.46 | 0.037 |
| Gefitinib | 1010 | 0.48 | 0.040 |

The coefficient reflects the change of drug sensitivity with the new 46-gene score in a cell line. The positive coefficient of a drug indicates that cell lines with higher 46-gene score were more sensitive to this drug.

**Table S7. Primer sequences used for qPCR.**

| **Gene** | **Primer sequence (5’-3’)** |
| --- | --- |
| Mouse *Gfap* | Forward: CAACGTTAAGCTAGCCCTGGACAT |
|  | Reverse: CTCACCATCCCGCATCTCCACAGT |
| Mouse *β-Actin* | Forward: CTGTCCCTGTATGCCTCTG |
|  | Reverse: ATGTCACGCACGATTTCC |
