# Supplementary material for: Forty‐three key gene expressions involved in the effect of indoleamine 2,3‐dioxygenase 1 expression on cancer prognosis may be a potential indoleamine 2,3‐dioxygenase 1 inhibitor biomarker
Source: Clin Transl Med. 2021 Feb 17;11(2):e330. doi: 10.1002/ctm2.330 (PMC7888544; doi:10.1002/ctm2.330)
Supplement: Supplementary file 2 — SuppMat2 [file CTM2-11-e330-s002.docx]

**Supporting Information 2:** Supplementary Figures

**43 key gene expressions involved in the effect of indoleamine 2,3-dioxygenase 1 (IDO1) expression on cancer prognosis may be a potential IDO1 inhibitor biomarker**

**Authors:** Weirui Li^1^, Leilei Guo^1^, Zikang Xing^1^, Xin Fang^1^, Heng liang^1^, Shengnan Zhang^1^, Lei Shi^1^, Chunxiang Kuang^2^, Leming Shi^1^, Yuanting Zheng^1^*, Yueqing Hu^1, 3^*, Qing Yang^1^*

*Corresponding author

**Author’s institutional affiliations:**

^1^ State Key Laboratory of Genetic Engineering, School of Life Sciences, Fudan University, Songhu Road 2005, Shanghai, 200438, China

^2^ Shanghai Key Lab of Chemical Assessment and Sustainability, School of Chemical Science and Engineering, Tongji University, 1239 Siping Road, 200092, Shanghai, China

^3^ Shanghai Center for Mathematical Sciences, Fudan University, Shanghai, China

Supplementary Figures


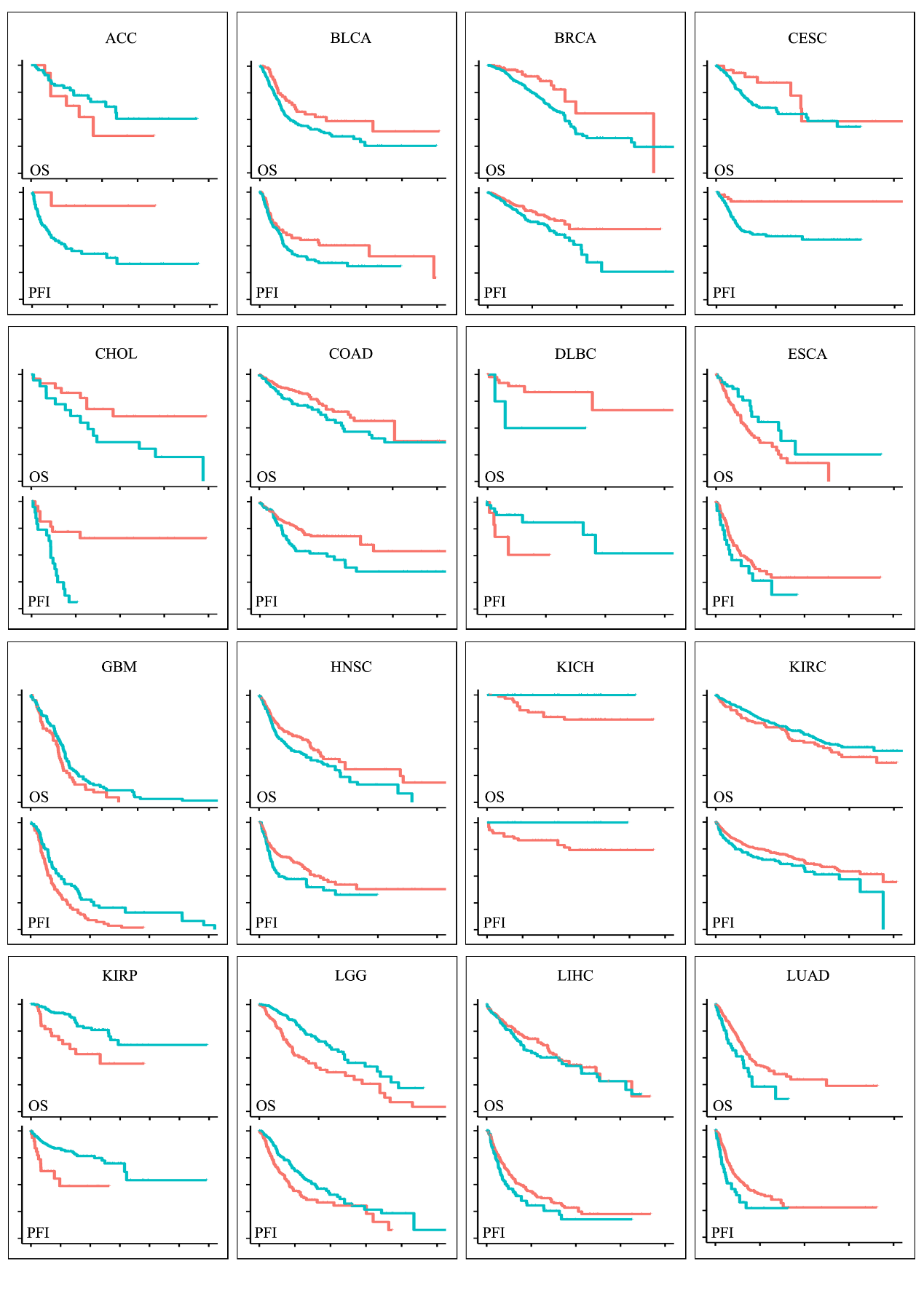
Figure S1


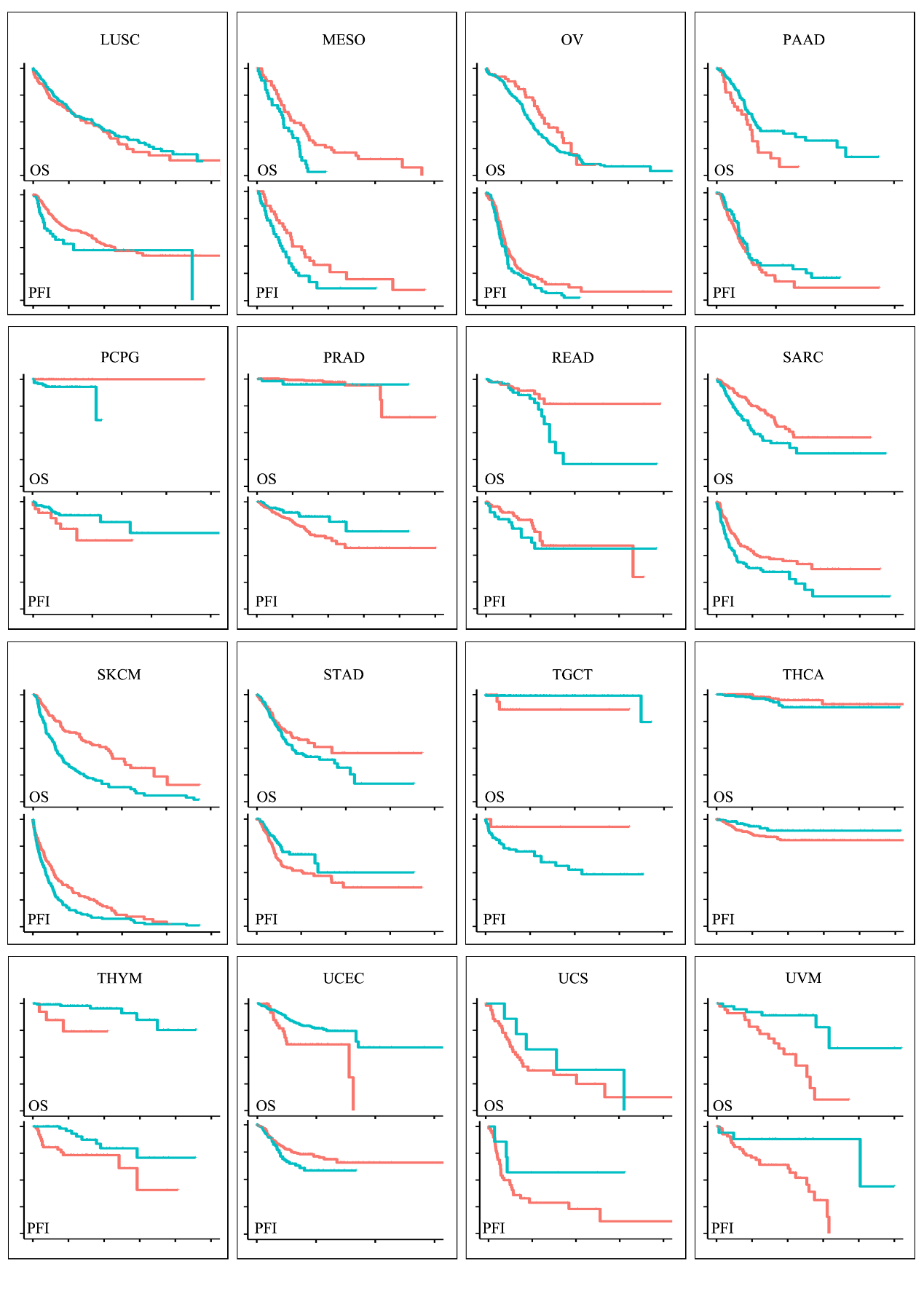

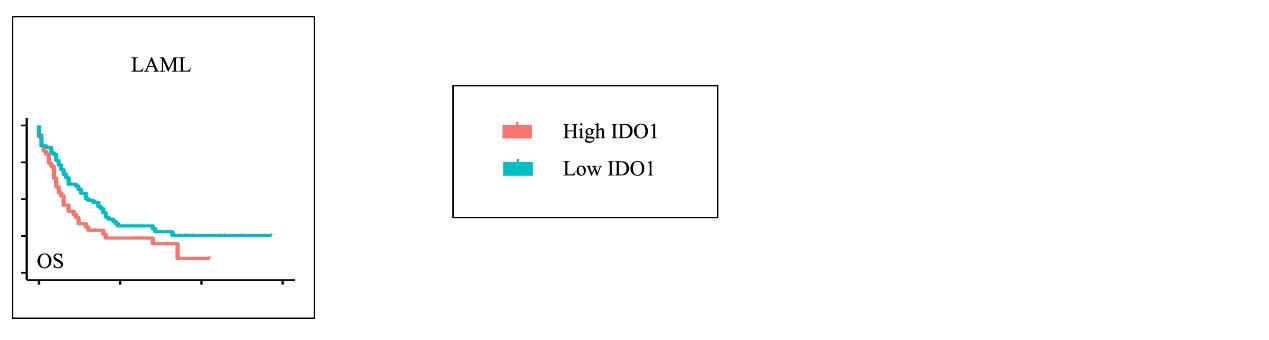


**Figure S1. Kaplan-Meier curves for patients stratified by the optimal cutoff of IDO1 mRNA expression in each of 33 cancers from TCGA. Refer to Table S2.**

The patients of LAML do not have PFI information. OS, overall survival; PFI, progression free interval.

Figure S2


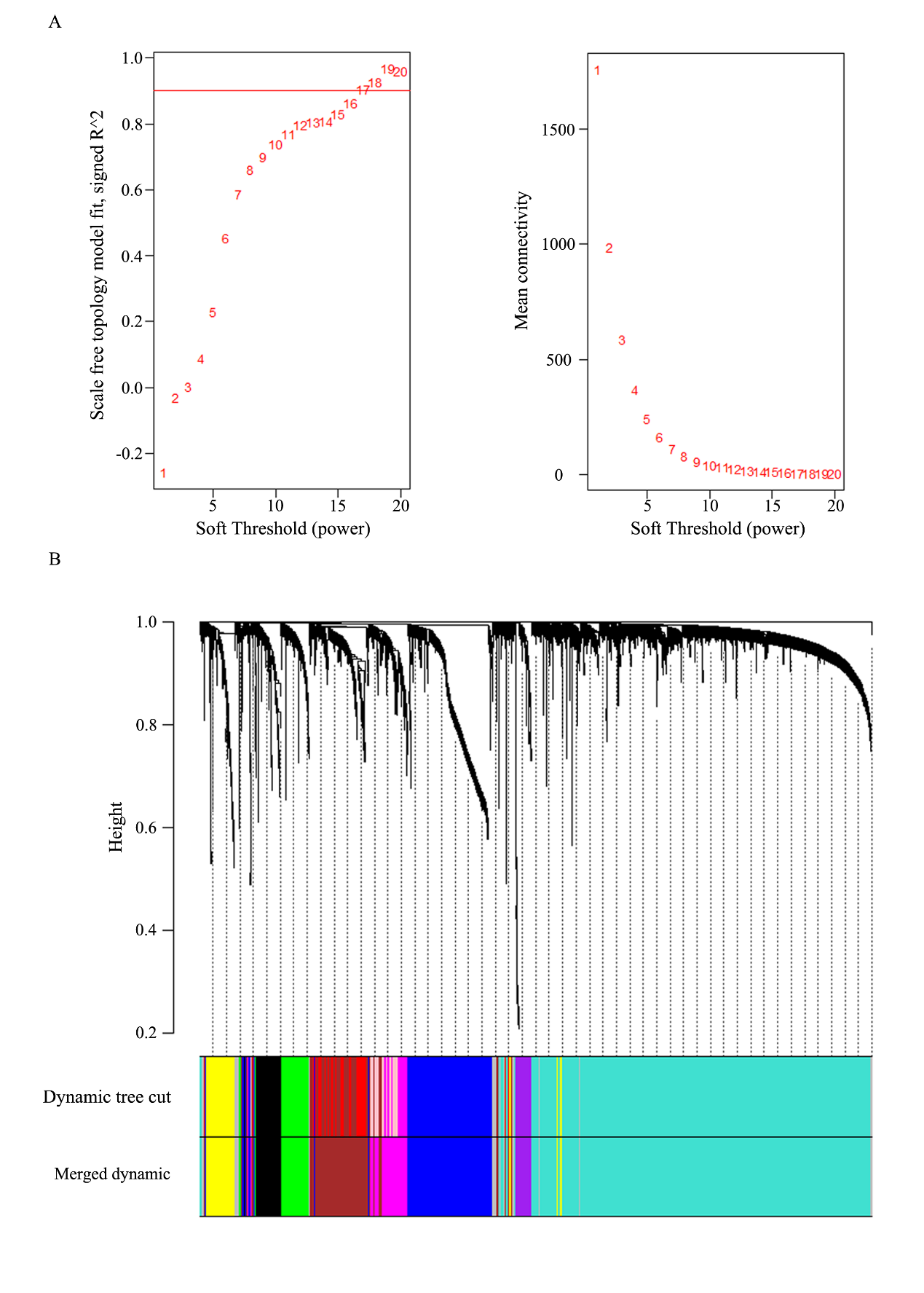


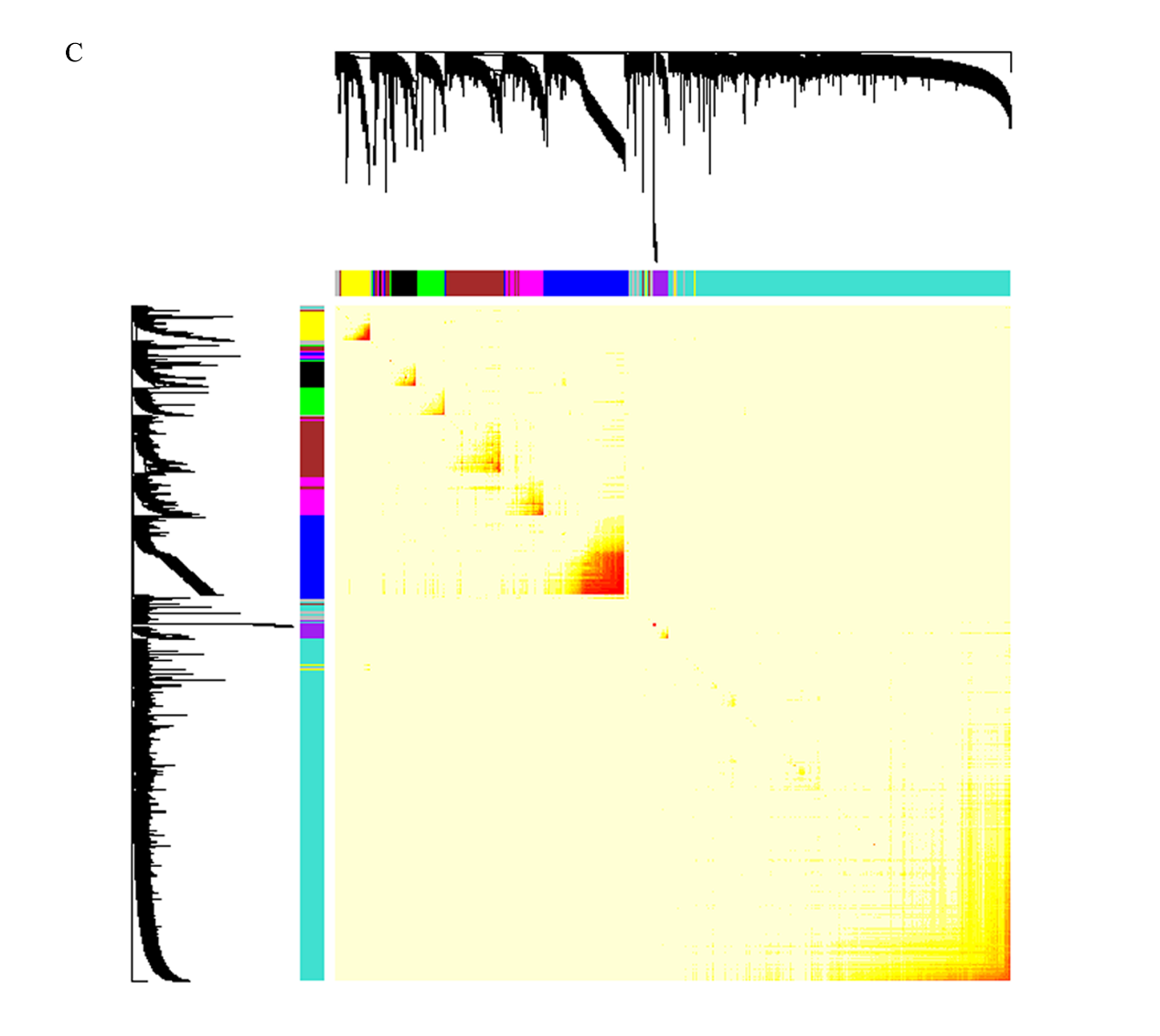


**Figure S2. Construction of co-expression modules of DEGs between patients in the IDO1 protective and deleterious categories.**

(A) The left and right panel showed the scale-free fit index and the mean connectivity for various soft-thresholding powers, respectively. When the soft-thresholding powers was equal to 18, the average degree of connectivity was close to zero. (B) The cluster dendrogram of 3375 DEGs between patients in the IDO1 protective and deleterious categories. Each branch in the figure represented one gene, and every color below represented one co-expression module. As a result, 8 co-expression modules were constructed and were shown in different colors. (C) Visualizing the gene network using a heatmap plot. The heatmap depicts the Topological Overlap Matrix among all genes in the analysis. Light color represents low overlap and progressively darker red color represents higher overlap. Blocks of darker colors along the diagonal are the modules. The gene dendrogram and module assignment are also shown along the left side and the top.

Figure S3


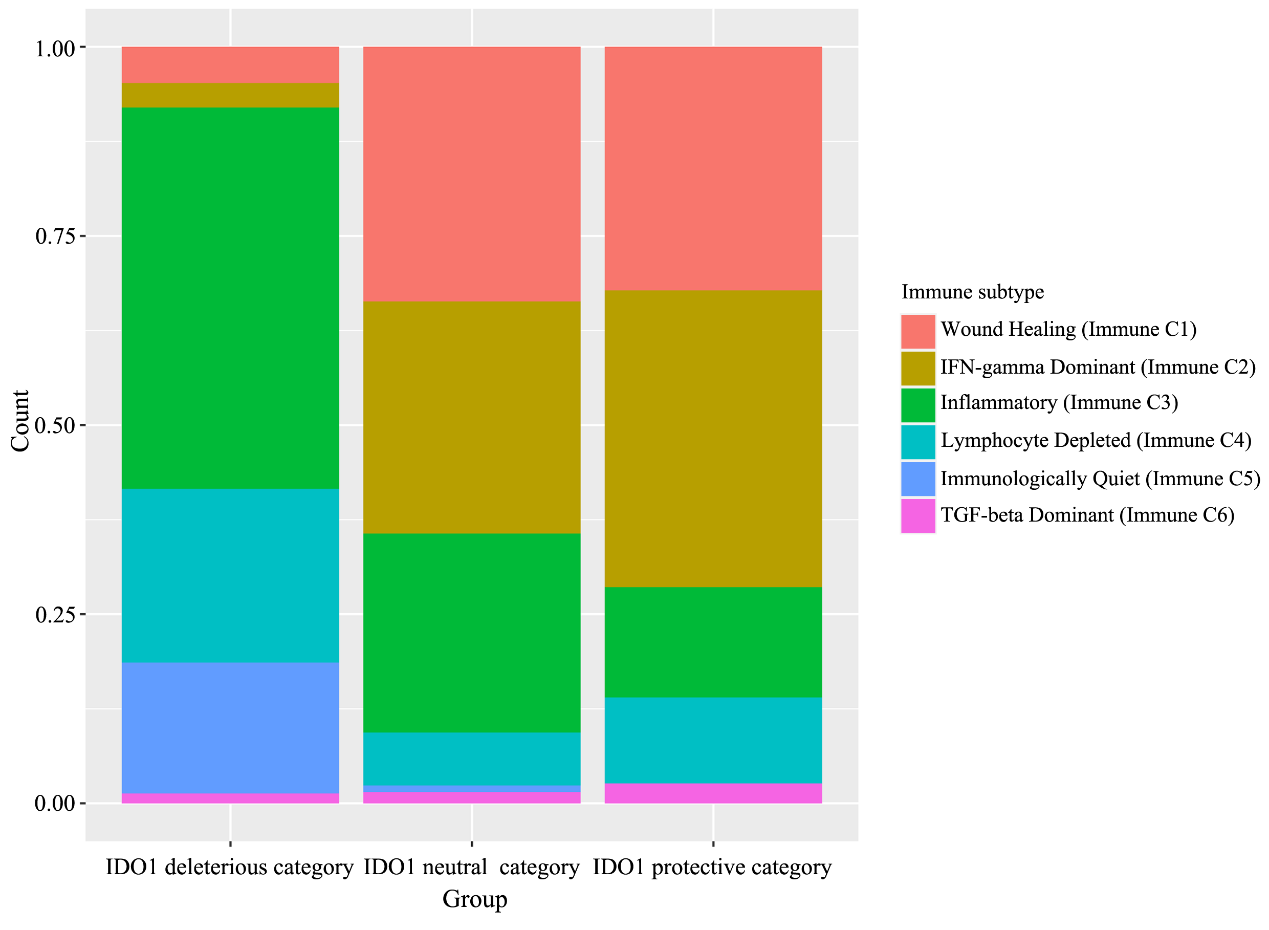


**Figure S3. Distributions of tumor immune subtype among patients in IDO1 deleterious, neutral, and protective categories.**

Percentage stacked histograms of 6 tumor immune subtypes in patients from IDO1 deleterious, neutral and protective categories. The proportion of 6 tumor immune subtypes in patients from IDO1 deleterious category was shown, which revealed the predominating proportion of tumor immune subtypes C3, C4, and C5 in these patients (left). The proportion of 6 tumor immune subtypes in patients from IDO1 neutral category was shown, which revealed the predominating proportion of tumor immune subtypes C1, C2, and C3 in these patients (middle). The proportion of 6 tumor immune subtypes in patients from IDO1 protective category was shown, which revealed the predominating proportion of tumor immune subtypes C1 and C2 in these patients (right).

Figure S4


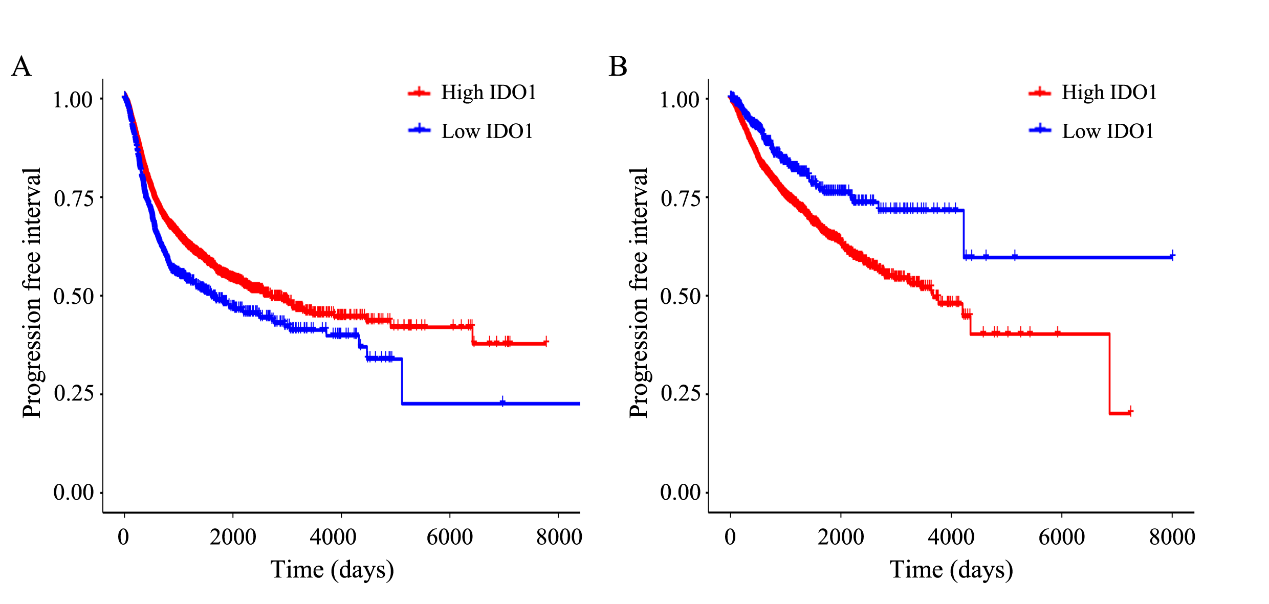


**Figure S4. Effect of *IDO1* on prognosis between patients with tumor immune subtypes C1 and C2 and patients with C3 and C5.**

(A) The Kaplan-Meier curve of PFI for different patients stratified by the optimal cutoff of IDO1 mRNA expression in patients with tumor immune subtypes C1 and C2. (B) The Kaplan-Meier curve of PFI for different patients stratified by the optimal cutoff of IDO1 mRNA expression in patients with tumor immune subtypes C3 and C5. PFI, progression free interval.

Figure S5


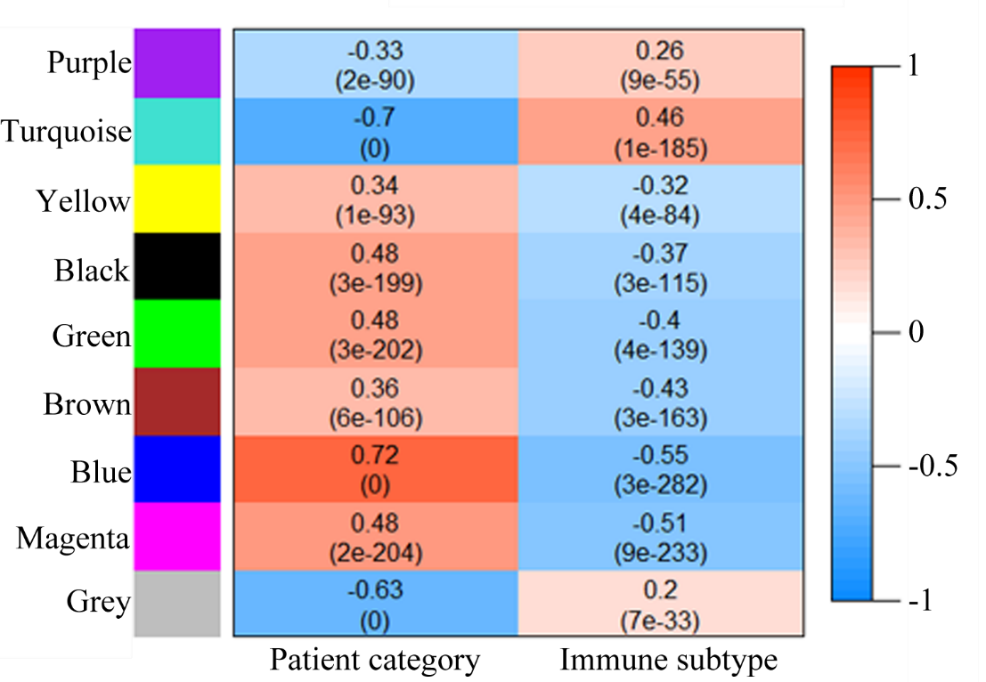


**Figure S5. Modules associated with tumor immune subtype and patient** **category.**

Heatmap of the correlation between tumor immune subtype, patient categorie and module eigengene. The color of cell in the heatmap represented the correlation coefficient of different size. Specifically, red color represented the positive correlation and blue color stood for the negative correlation. The figure without bracket in each cell indicated the correlation coefficient of clinical features. The corresponding p-value was shown in parentheses. The turquoise, blue, green and magenta module were significantly correlated with tumor immune subtype and patient category.

Figure S6


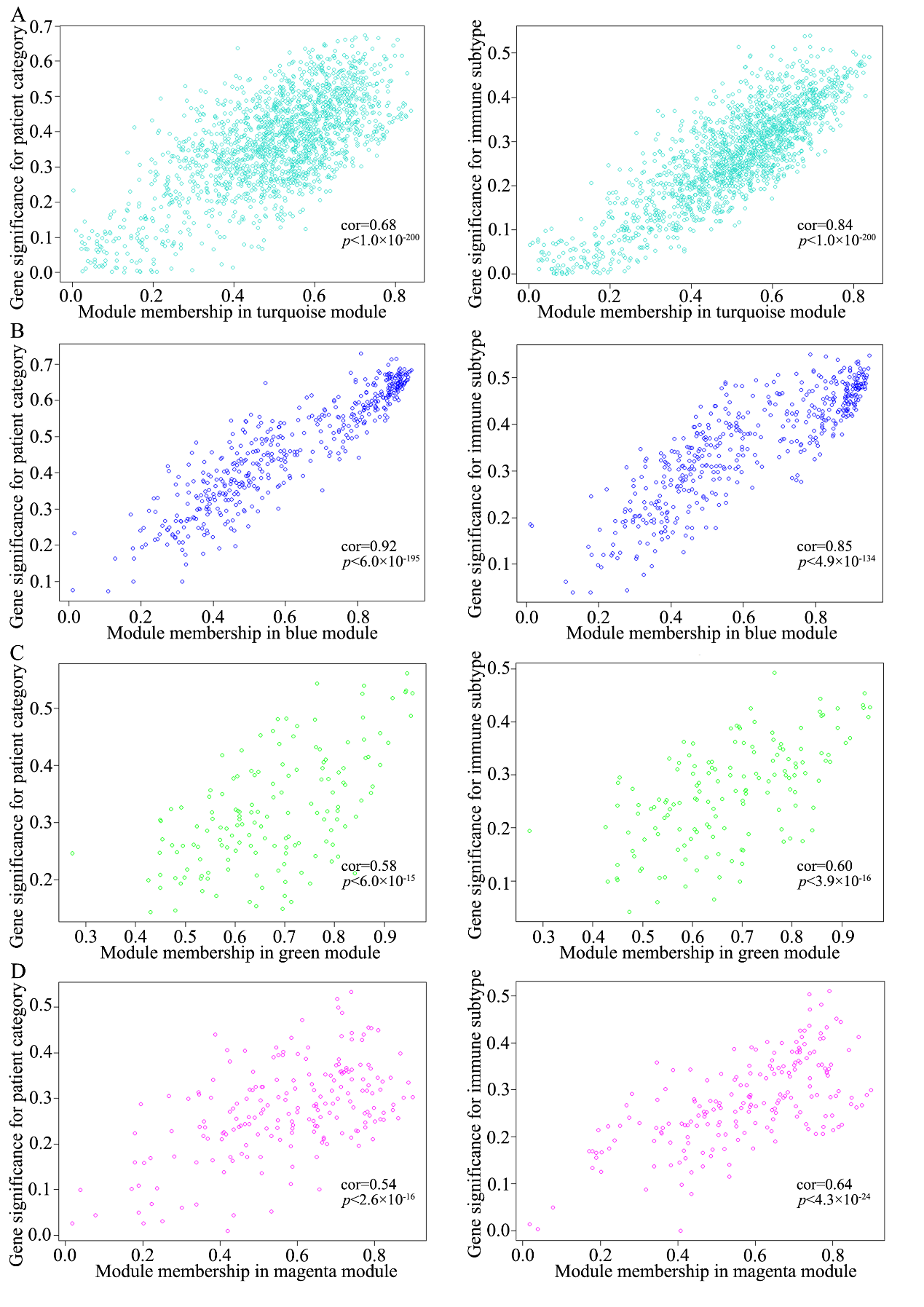


**Figure S6.** **Strong correlation between gene significance and module membership in** **turquoise, blue, green and magenta modules.**

(A) Scatterplots of Gene Significance for patient category (left) vs. Module Membership (MM) and tumor immune subtype (right) vs. MM in the turquoise module. (B) Scatterplots of Gene Significance for patient category (left) vs. MM and tumor immune subtype (right) vs. MM in the blue module. (C) Scatterplots of Gene Significance for patient category (left) vs. MM and tumor immune subtype (right) vs. MM in the green module. (D) Scatterplots of Gene Significance for patient category (left) vs. MM and tumor immune subtype (right) vs. MM in the magenta module.

Figure S7


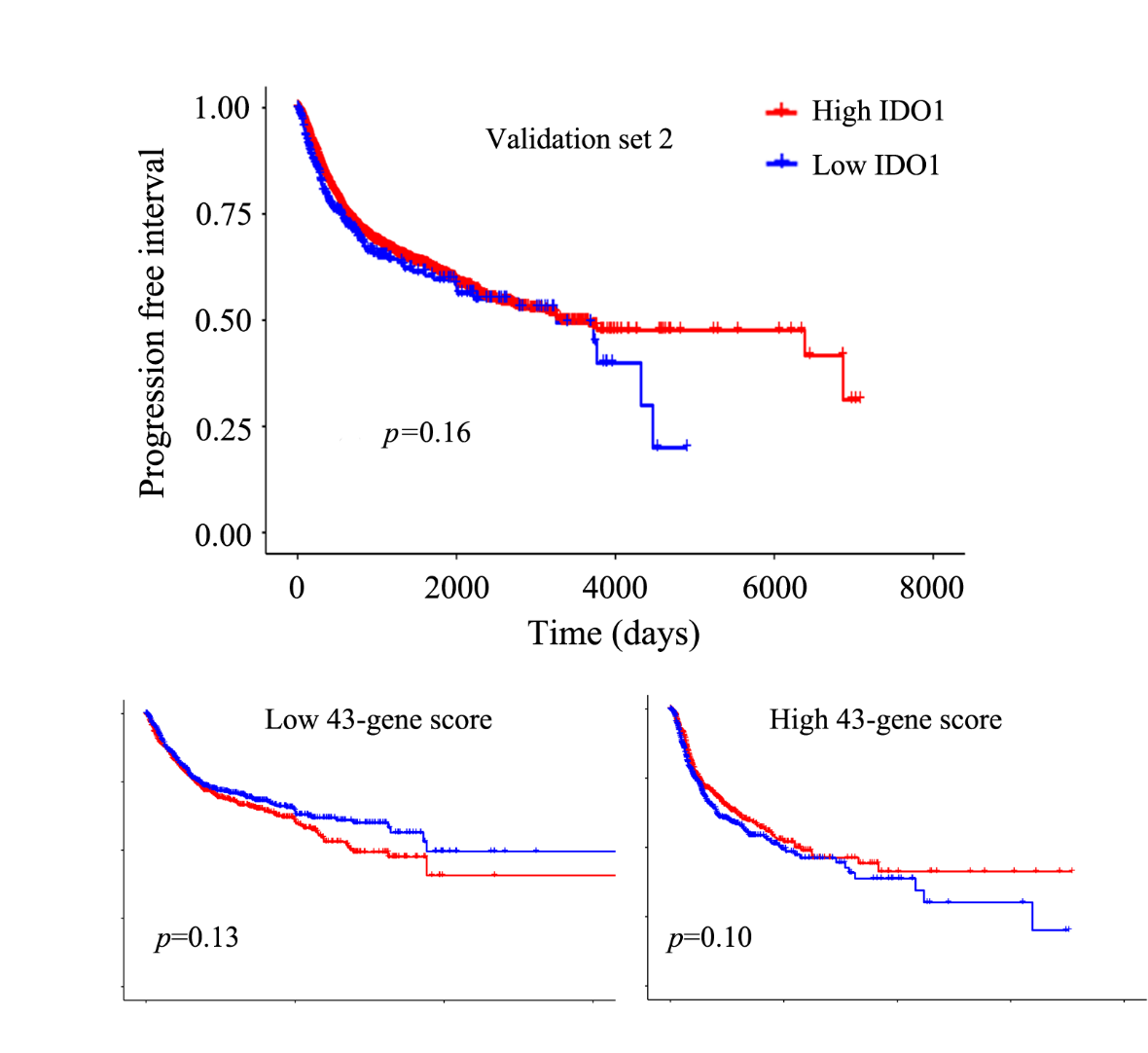


**Figure S7. Poor prediction power of 43-gene score in the validation set 2.**

# Figure S8


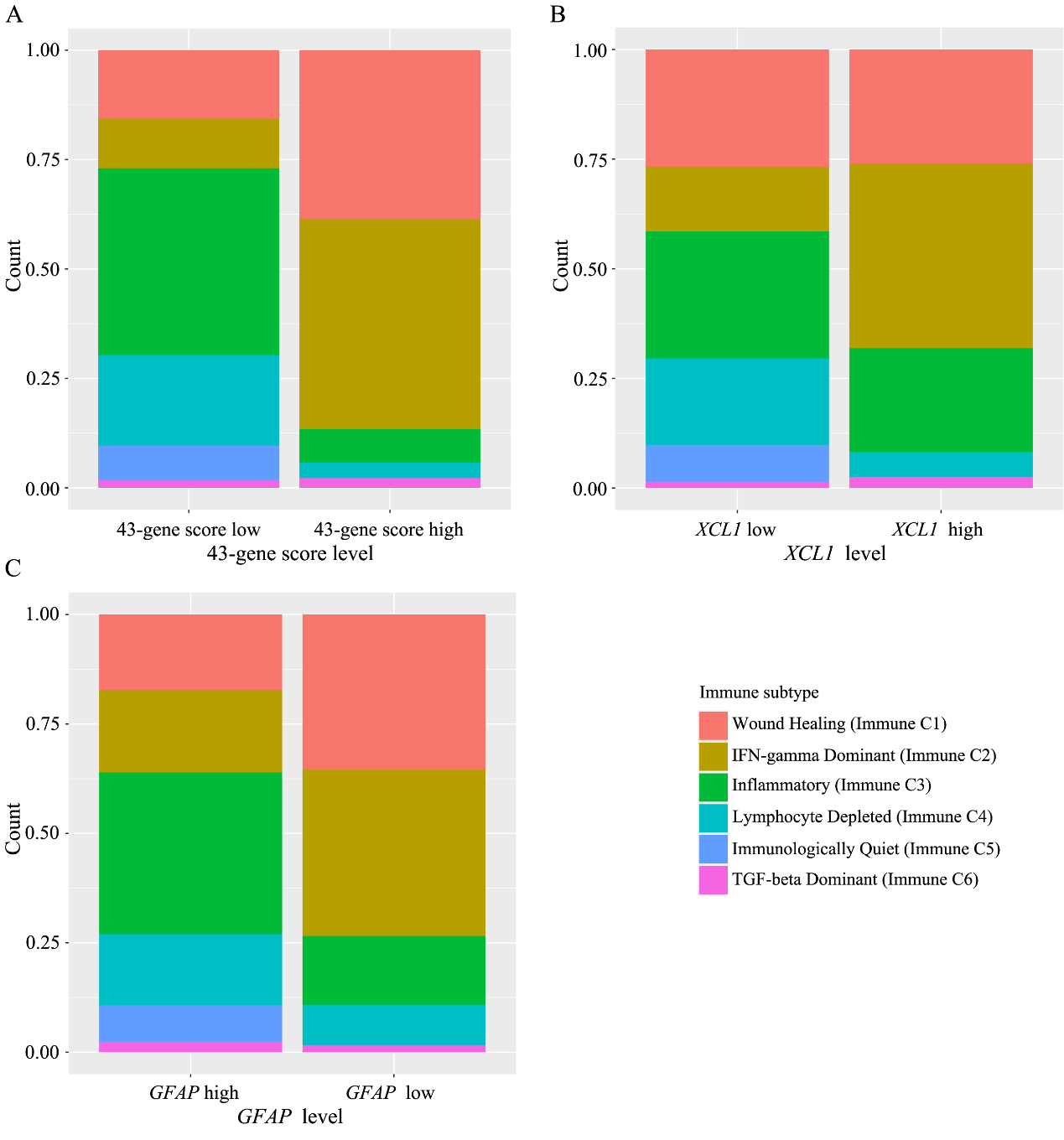


**Figure S8. Distribution of tumor immune subtype in patients with high/low 43-gene score, *XCL1* and *GFAP* expression levels.**

(A) Percentage stacked histograms of 6 tumor immune subtypes in patients with high and low 43-gene score levels. (B) Percentage stacked histograms of 6 tumor immune subtypes in patients with high and low *XCL1* levels. (C) Percentage stacked histograms of 6 tumor immune subtypes in patients with high and low *GFAP* levels.

# Figure S9

**
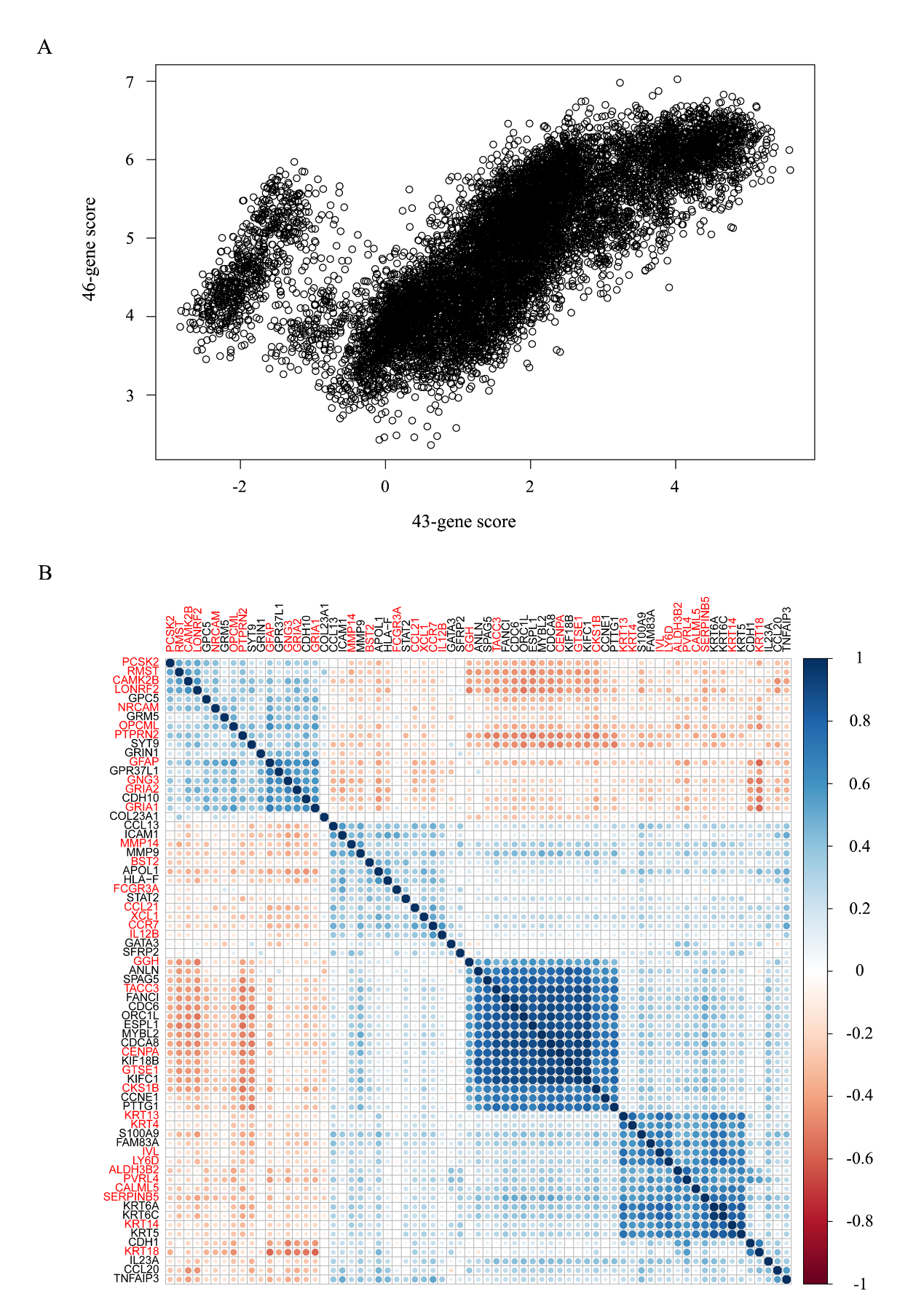
**

**Figure S9. Correlations between old and new key genes.**

We randomly selected 4408 patients from IDO1 protective and deleterious categories as the new discovery set, performed the same procedure as described in the text, and then got 46 new key genes. The old key genes are genes contained in 43 key genes and not contained in 46 new key genes. The new genes are genes not contained in 43 key genes and contained in 46 new key genes. (A) 46-gene score is the weighted average of 46 new key gene expressions and 43-gene score is the weighted average of 43 key gene expressions. The scatter plot shows a strong correlation between 46-gene scores and 43-gene scores in all patients in the TCGA Pan-Cancer dataset. (B) Correlation heat map of expression of different genes between 46 new key genes and 43 key genes. There is a strong correlation between old (red) and new (black) key genes.
