# Supplementary material for: Forty‐three key gene expressions involved in the effect of indoleamine 2,3‐dioxygenase 1 expression on cancer prognosis may be a potential indoleamine 2,3‐dioxygenase 1 inhibitor biomarker
Source: Clin Transl Med. 2021 Feb 17;11(2):e330. doi: 10.1002/ctm2.330 (PMC7888544; doi:10.1002/ctm2.330)
Supplement: Supplementary file 3 — SuppMat3 [file CTM2-11-e330-s003.docx]

**Supporting Information 3:** Materials and methods

**43 key gene expressions involved in the effect of indoleamine 2,3-dioxygenase 1 (IDO1) expression on cancer prognosis may be a potential IDO1 inhibitor biomarker**

**Authors:** Weirui Li^1^, Leilei Guo^1^, Zikang Xing^1^, Xin Fang^1^, Heng liang^1^, Shengnan Zhang^1^, Lei Shi^1^, Chunxiang Kuang^2^, Leming Shi^1^, Yuanting Zheng^1^*, Yueqing Hu^1, 3^*, Qing Yang^1^*

*Corresponding author

**Author’s institutional affiliations:**

^1^ State Key Laboratory of Genetic Engineering, School of Life Sciences, Fudan University, Songhu Road 2005, Shanghai, 200438, China

^2^ Shanghai Key Lab of Chemical Assessment and Sustainability, School of Chemical Science and Engineering, Tongji University, 1239 Siping Road, 200092, Shanghai, China

^3^ Shanghai Center for Mathematical Sciences, Fudan University, Shanghai, China

**Materials and methods**

**1 Patient samples from TCGA**

More than 11000 patients of 33 diverse types of cancers were included in this study. The mRNA expression data, clinical information and immune subtype data of those patients were from TCGA Pan-Cancer (download from https://xenabrowser.net/datapages/). The source datasets were harmonized by the Pan-Cancer Atlas consortium for uniform quality control, batch effect correction and normalization^1^.

11014 samples having mRNA expression data and the corresponding clinical data were used for the survival analysis. Of those, 5644 samples were from female ones and 5370 samples were from male ones, and 9104 samples had their own immune subtype information. The age of initial pathological diagnosis ranged from 14 to 90 years with a median of 61.

**2 Survival analysis**

The R package survival (version 2.43-3) was employed to conduct the association study between *IDO1* expression and prognosis by using Kaplan-Meier method and Cox Proportional Hazards (CoxPH) regression model. The R package survminer (version 0.4.6) was used to determine the optimal cutoff of *IDO1* mRNA expression based on the minimum *p*-value approach^2^. *IDO1* mRNA expression > optimal cutoff was regarded as high expression, and < optimal cutoff as low expression. Following the recommendations of a specialized study^3^, PFI and OS were the clinical endpoints in the subsequent survival analysis. The log-rank test and likelihood ratio test were used to calculate the *p*-value of Kaplan-Meier method and in fitting CoxPH regression model, respectively. *P*-value < 0.05 was considered as statistically significant.

Note that we followed the two rules as described below in combining the outcomes of survival analyses of clinical endpoints OS and PFI. Firstly, for a given type of cancer, if only one of OS and PFI was recommended^3^, then the association between *IDO1* expression and prognosis in terms of this endpoint will be reported. Secondly, if both two endpoints were recommended for use, but the corresponding outcomes are not consistent, *IDO1* expression will be considered to not correlate to prognosis for this type of cancer.

In studying the association between *IDO1* expression and prognosis for patients with 33 different cancer types, the *IDO1* mRNA expression was standardized within each type of cancer, so as to eliminate the interference of varied cancer types on the *IDO1* expression or prognosis. Based on the statistical analysis of patients’ survival information, all patients are classified into 3 categories of IDO1 protective, deleterious, and neutral.

**3 Differentially expressed genes (DEGs) between patients in IDO1 protective and deleterious categories**

The R package limma^4^ (version 3.38.3) was used to detect DEGs between two groups of patients. Student’s t-test was used to determine the significance of the difference (*p*-value) of each gene expression. Multiple hypothesis testing correction was conducted using the Benjamini and Hochberg false discovery rate^5^, and the adjusted *p*-values were then calculated. DEGs were filtered based on the threshold of the adjusted *p*-values < 0.01 and |log_2_ fold change (FC)| > 1.

**4 Weighted gene co-expression network analysis**

The R package WGCNA^6^ (version 1.68) was used for construction of weighted gene co-expression network. The parameters used in the network construction were determined as follows. First, the soft threshold power of 18 was selected according to the requirements of scale-free network^7^. This soft threshold determines the adjacency matrix of the network. Then, the minClusterSize and deepSplit parameters were set to 20 and 2, respectively, in the determination of initial co-expression modules by cutreeDynamic function. Finally, the module eigengene (ME) of each module, which represents the expression level for each module, was calculated. The modules with ME correlation coefficient being greater than 0.8 were merged to obtain the final co-expression modules.

Patient category, which has three different patterns in terms of the effect of *IDO1* on prognosis, and immune subtype are of our interest. The correlation coefficients between ME and patient category, ME and immune subtype in every module were also calculated by the R package WGCNA. The modules with these two correlation coefficients being greater than 0.4 were considered to be associated with patient category and immune subtype.

**5 Gene set enrichment analysis**

Gene set enrichment analysis (GSEA) was used to investigate the enriched gene ontology (GO) terms and Kyoto Encyclopedia of Genes and Genomes (KEGG) pathways, and find the leading edge genes. GSEA is first to rank genes based on the extent of difference of gene expression between two types of samples, and then test whether a preselected genes set are enriched at the top or bottom of the ranking list. In this study, the ranking list was obtained according to FC. The R package clusterProfiler^8^ (version 3.10.1) was used to conduct GSEA. The number of gene set permutations for each analysis was fixed at 1000. As above, Benjamini and Hochberg false discovery rate was used for multiple testing adjustment. GO terms and KEGG pathways with adjusted *p*-values < 0.05 were considered as significantly enriched gene sets.

Leading edge genes were determined as ones in the gene set that appear in the ranked list at or before the point where the running sum reaches its maximum deviation from zero, acting as the core of a gene set that accounts for the enrichment signal. Leading edge genes in each significantly enriched gene set were provided by GSEA.

**6 Protein‐protein interaction network analysis**

The protein‐protein interaction network (PPIN) was constructed by the Search Tool for the Retrieval of Interacting Genes/Proteins (STRING) online database^9^ (version 11.0). The PPIN was visualized using Cytoscape software^10^ (version 3.7.0). Let *n_F_* be the number of candidate genes detected in either of the four steps described in sections 3-6 of Materials and methods.

**7 Random-forest-based variable importance measure**

The expression of the *n_F_* candidate genes above mentioned and patient category (protective or deleterious) were used as the optional variables in growing the random forest to find out which variables influenced the association between *IDO1* expression and prognosis in the discovery set. For each splitting in a tree, we selected the variable which can classify the patients into two groups with the greatest difference of hazard ratios (HRs) of *IDO1* high expression and prognosis. For example, HRs of *IDO1* high expression in the two sub nodes splitting with variable V1 are respectively 1.2 and 0.8, and the HRs of *IDO1* high expression in the two sub nodes splitting with variable V2 are respectively 1.5 and 0.6. Variable V2 will be selected for this splitting because 0.9 (1.5 minus 0.6) is greater than 0.4 (1.2 minus 0.8).

The more times a variable is selected for splitting, the more important the variable is, and the more significant its effect on the association between *IDO1* expression and prognosis is. For the null hypothesis that every variable is randomly selected with equal probability for splitting, i.e. no variables have an effect on the association between *IDO1* expression and prognosis, we can infer the times that a variable is selected for splitting follows a binomial distribution.

Let *N_F_* be the number of trees in a random forest and *s* be the splitting times for every tree therein. Under the null hypothesis that each of the *n_F_* +1 variable is equally likely to be chosen as the splitting variable in the formation of the two sub-nodes, we can use a Bernoulli random variable to describe the time of a given variable being selected for splitting. Based on the property of independent Bernoulli random variables, the times of a given variable being used in the construction of a random forest having *N_F_* trees follows binomial (*N_F_*$*$*s*, 1/(*n_F_* +1)).

When the *p*-value calculated from the binomial distribution is less than a given threshold, the null hypothesis is rejected, and the variable has influence on the association between *IDO1* expression and prognosis. In the present study, the parameter *N_F_* was 300, *n_F_* was 540, and *s* was 3. For a given threshold of 0.01, we concluded a set of 43 key genes being selected for splitting more than 5 times in the random forest.

**8 43-gene score**

For every gene *i* of those 43 genes, 1 ≤ *i* ≤ 43, if its mean expression in the patients of IDO1 protective category is bigger than that of deleterious category within the discovery set, let *w_i_* = 1, and -1 otherwise. For any patient, let *E*_i_ be the expression of gene *i*，and we define $\sum_{i=1}^{43} w_{i}*E_{i}/$43 as the 43-gene score for the subsequent analysis.

**9 High 43-gene score cell lines sensitive drugs**

We downloaded the efficacy measures of 251 drugs across 983 human cancer cell lines from the Genomics of Drug Sensitivity in Cancer (GDSC) database (ftp://ftp.sanger.ac.uk/pub/project/cancerrxgene/releases/release-7.0/) and used them to identify drugs which cell lines with higher 43-gene scores are more sensitive to. We used the same criteria as in Jerby-Arnon et al.^11^ to convert IC_50_ and 43 gene scores into binary variables. For each drug, cell lines with the lowest (bottom 25%) IC_50_ values were defined as sensitive ones. The gene expression was used to compute the 43-gene score for each of the 983 cells. The cell lines with the highest 43-gene scores (top 25%) were defined as IDO1 blocking ‘‘resistance’’ ones. For each drug, the R package lme4 was employed to build a hierarchical logistic regression model, where the dependent variable is the cell line’s (drug-specific) binary sensitivity assignment, and the independent variables are the cell line’s ‘‘resistance’’ assignment (level-1) and patient category (level-2). The output *p*-values and coefficients quantified the association between the drug sensitivity (dependent) variable and the IDO1 blocking resistance (independent) variable.

**10 Cell culture and treatment**

The mouse Lewis lung carcinoma (LLC) cell line was purchased from the American Type Culture Collection (ATCC) and were maintained in Dulbecco's modified Eagle’s medium (DMEM, Gibco, USA) containing 10% fetal bovine serum (Gibco, USA) and 1% penicillin-streptomycin (Gibco, USA). Cells were grown at 5% CO2 in a humidified incubator at 37°C.

LLC cells were exposed to the following conditions: Gefitinib, incubated with 100 nM gefitinib (Iressa, Aladdin, China) in DMEM which contained 0.01% dimethyl sulfoxide (DMSO, Sinopharm, China) for 96 h. Control, incubated with 0.01% DMSO in DMEM for 96 h.

**11 LLC tumor-bearing mice and treatments**

Female, 6-week-old, C57BL/6 mice were purchased from SLAC Experimental Animal Center, Shanghai, China. The experimental procedures were approved by the Animal Ethics Committee of Fudan University and performed in compliance with ARRIVE guidelines. IDO1 inhibitor RY103 was developed by our lab. IDO1 inhibitor L-1-MT was purchased from Sigma.

1 × 10^6^ LLC cells were subcutaneously injected into the right flank of mice. Five days after the injection, the LLC tumor-bearing mice were divided randomly into nine groups: control group, INCB024360 group, gefitinib group, L-1-MT group, gefitinib plus L-1-MT group, gefitinib followed by L-1-MT group, RY103 group, gefitinib plus RY103 group and gefitinib followed by RY103 group. Gefitinib followed by IDO1 inhibitor groups initiated administration on 7^th^ day after the injection and other groups initiated administration on 9^th^ day. Treatments lasted until the 18^th^ day after the injection, control group, INCB024360 group and gefitinib group respectively received 0.5% sodium carboxymethyl cellulose (CMC-Na), 50 mg/kg INCB024360 and 50 mg/kg gefitinib in 0.5% CMC-Na, i.g. per 24 h; L-1-MT group and RY103 group respectively received 100 mg/kg L-1-MT in 10% hydroxypropyl-β-cyclodextrin (HPBCD), i.p. per 24 h and 6 mg/kg RY103 in 10% HPBCD, i.p. per 36 h; Combo group including gefitinib plus L-1-MT group and gefitinib plus RY103 group received 50 mg/kg gefitinib in 0.5% CMC-Na, i.g. per 24 h, plus 100 mg/kg L-1-MT in 10% HPBCD, i.p. per 24 h or 6 mg/kg RY103 in 10% HPBCD, i.p. per 36 h. From the 7^th^ day to the 18^th^ day after the injection, the gefitinib followed by L-1-MT group and gefitinib followed by RY103 group first received 50 mg/kg gefitinib in 0.5%CMC, i.g. per 24 h for three days, and respectively received 100 mg/kg L-1-MT in 10% HPBCD, i.p. per 24 h and 6 mg/kg RY103 in 10% HPBCD, i.p. per 36 h since the 9^th^ day. Mice were sacrificed in the 19^th^ days after the injection. The blood and tumors were collected.

**12 RT-PCR and quantitative real-time PCR (qPCR)**

Total RNA was extracted from LLC cells or tumors using TRIzol Reagent (Invitrogen, USA). RT-PCR was performed to synthesize cDNA using a PrimeScript^TM^ RT Master Mix kit (Takara, Japan). qPCR was performed in triplicate to detect the expression levels of *Gfap* and *β-Actin* using the AceQ^®^ qPCR SYBR Green Master Mix kit (Vazyme, China). *β-Actin* was used as the internal control. The amplification program consisted of activation at 95°C for 5 min, followed by 40 amplification cycles consisting of 95°C for 10 s, 60°C for 30 s, and elongation at 95°C for 15 s, 60°C for 60 s, 95°C for 15 s. The primers used for the qPCR were shown in Table S7. Data were analyzed using My IQ software (Bio-Rad, Germany).

**13 Analysis of IDO1 activity in serums**

The IDO1 activity was evaluated by measuring the levels of Trp and Kyn by HPLC and calculating the Kyn/Trp ratio^12^.

Blood samples were collected in lithium heparin or ethylene diamine tetraacetic acid vacutainer venous blood collection tubes. The serums were separated from blood samples by centrifugation at 3000 g for 15 min and stored at -80°C.

The serums were treated with 5% perchloric acid and methanol to remove protein, and the supernatants were subjected to HPLC analysis. The analysis was performed on an Agilent 1260 series HPLC system (Agilent Technologies, USA) equipped with a quaternary pump and a UV detector. HPLC analysis of the samples was performed using an Agilent C18 column (5 μm particle size, L × I.D. 25 cm × 4.6 mm) preceded by a C18 guard column (Dikma, China). The mobile phase (pH 3.6) consisted of 15 mM acetic acid-sodium acetate buffer and acetonitrile at a ratio of 94:6. Flow rate is 1 mL/min. The detected wavelengths were 280 nm for Trp and 360 nm for Kyn.

**14 Statistical analyses**

The distributions of immune subtype between or among different patient categories were compared by chi-square test. The distributions of 43-gene score in IDO1 deleterious and protective categories were compared by Mann-Whitney U test.

The data of in *vitro* and in *vivo* experiments were plotted by GraphPad Prism 6.0 software, and expressed as mean ± standard error (mean ± SEM). The comparison between two groups was conducted by Student's t-test. One-way analysis of variance (ANOVA) followed by Dunnett's multiple comparisons test was used to compare several treatment groups with a control group.

The “*n*” in the figure legends indicates the number of patients, mice or independent cell culture preparations.

**Reference**

1. Ellrott K, Bailey MH, Saksena G, Covington KR, Kandoth C, Stewart C, et al. Scalable open science approach for mutation calling of tumor exomes using multiple genomic pipelines. *Cell Syst.* 2018;6(3):271-281.
2. Mazumdar M, Smith A, Bacik J. Methods for categorizing a prognostic variable in a multivariable setting. *Stat Med.* 2003;22(4):559-571.
3. Liu J, Lichtenberg T, Hoadley KA, Poisson LM, Lazar AJ, Cherniack AD, et al. An integrated TCGA pan-cancer clinical data resource to drive high-quality survival outcome analytics. *Cell.* 2018;173(2):400-416.
4. Smyth GK, Michaud J, Scott HS. Use of within-array replicate spots for assessing differential expression in microarray experiments. *Bioinformatics.* 2005;21(9):2067-2075.
5. Benjamini Y, Drai D, Elmer G, Kafkafi N, Golani I. Controlling the false discovery rate in behavior genetics research. *Behav Brain Res.* 2001;125(1-2):279-284.
6. Langfelder P, Horvath S. WGCNA: An R package for weighted correlation network analysis. *BMC Bioinformatics.* 2008;9:559.
7. Zhang B, Horvath S. A general framework for weighted gene co-expression network analysis. *Stat Appl Genet Mol Biol.* 2005;4:Article17.
8. Yu G, Wang LG, Han Y, He QY. Clusterprofiler: An R package for comparing biological themes among gene clusters. *OMICS.* 2012;16(5):284-287.
9. Szklarczyk D, Morris JH, Cook H, Kuhn M, Wyder S, Simonovic M, et al. The STRING database in 2017: Quality-controlled protein-protein association networks, made broadly accessible. *Nucleic Acids Res.* 2017;45(D1):D362-D368.
10. Su G, Morris JH, Demchak B, Bader GD. Biological network exploration with Cytoscape 3. *Curr Protoc Bioinformatics.* 2014;47:8 13 11-24.
11. Jerby-Arnon L, Shah P, Cuoco MS, Rodman C, Su MJ, Melms JC, et al. A cancer cell program promotes T cell exclusion and resistance to checkpoint blockade. *Cell.* 2018;175(4):984-997.
12. Adams S, Teo C, McDonald KL, Zinger A, Bustamante S, Lim CK, et al. Involvement of the kynurenine pathway in human glioma pathophysiology. *PLoS One.* 2014;9(11):e112945.
